# Deletion of *ppe27* in *Mycobacterium bovis* BCG augments macrophage immune responses during infection

**DOI:** 10.1128/spectrum.00915-26

**Published:** 2026-10-06

**Authors:** Tz-Chun Guo, Shintaro Seto, Minako Hijikata, Naoto Keicho

**Affiliations:** 1 Department of Pathophysiology and Host Defense, The Research Institute of Tuberculosis, Japan Anti-Tuberculosis Association 91331, Tokyo, Japan; 2 The Research Institute of Tuberculosis, Japan Anti-Tuberculosis Association 91331, Tokyo, Japan; University of Hawaii at Manoa, Honolulu, Hawaii, USA; Sichuan University, Chengdu, China

**Keywords:** *Mycobacterium tuberculosis*, *Mycobacterium bovis *BCG, ESX-5, PE/PPE, PPE27, macrophages, RNA sequencing

## Abstract

Mycobacterial infection induces dynamic transcriptional changes in both the host and pathogen. In slow-growing mycobacteria, the ESX-5 secretion system plays a crucial role in virulence by exporting Pro–Glu (PE) and Pro–Pro–Glu (PPE) proteins, which modulate host immune responses and contribute to pathogenesis. Previous studies demonstrated that *ppe27* overexpression in *Mycobacterium smegmatis* enhances bacterial survival and promotes host cell death, suggesting that PPE27 plays an important role in resistance to macrophage-induced cytotoxicity. However, its function during host–pathogen interactions remains poorly defined. To elucidate the function of PPE27, we generated a *ppe27* deletion mutant of *Mycobacterium bovis* Bacillus Calmette–Guérin (BCG∆*ppe27*) and performed RNA-seq to simultaneously profile host and bacterial transcriptional responses during macrophage infection. Host transcriptome analysis revealed that *ppe27* deletion led to upregulated pro-inflammatory responses, including enhancement of the TNF-α, IFN-α, and IL-6 signaling pathways. In parallel, bacterial transcriptome analysis identified downregulation of *JTY_3006* in BCG∆*ppe27* during infection compared with its expression in wild-type BCG. Amino acid sequence analysis indicated that *JTY_3006* shares 100% identity to a S-adenosylmethionine (SAM)-dependent methyltransferase conserved among mycobacterial species. Taken together, these findings suggest that PPE27 may contribute to the negative modulation of host inflammatory responses and suggest that manipulation of PPE27 might reshape pathogenicity and inform future vaccine strategies.

**IMPORTANCE:**

Tuberculosis remains the leading cause of death from a single infectious agent worldwide. Understanding the mechanisms that allow *Mycobacterium tuberculosis* to evade host defenses is essential for developing better vaccines, therapeutics, and prevention strategies. This study focuses on PPE27, a member of the PE/PPE protein family secreted through the ESX-5 secretion system. Although PE/PPE proteins are known to trigger strong immune responses, their precise role in pathogenesis has been unclear. Our study reveals that *ppe27* deletion is associated with enhanced macrophage inflammatory responses. Understanding this mechanism provides valuable insight into host–pathogen interactions and offers new directions for tuberculosis vaccine and therapeutic development.

## INTRODUCTION

Tuberculosis causes an estimated 10.7 million incident cases and approximately 1.23 million deaths in 2024 (1), underscoring the urgent need to better understand *Mycobacterium tuberculosis* virulence mechanisms and host–pathogen interactions to develop a more effective intervention strategy. The long-lasting co-evolution of *M. tuberculosis* and humans has shaped their interplay, with bacterial adaptations that enhance infectivity being counteracted by corresponding changes in host defense mechanisms (2).

Upon entry into host macrophages, *M. tuberculosis* components are sensed by pattern recognition receptors, triggering signaling pathways that regulate phagocytosis, autophagy, apoptosis, inflammasome activation, and cytokine production (3). In parallel, *M. tuberculosis* survives inside macrophages by subverting host immunity, specifically by using diverse lipids and secreted proteins to arrest phagosome maturation and manipulate cell death pathways, thereby enhancing intracellular survival and dissemination. Central to these processes are type VII secretion systems (T7SSs), particularly the ESX family. T7SSs export virulence factors that modulate host immunity and sabotage antimicrobial defenses. Mycobacteria possess up to five homologous T7SS clusters (ESX-1–5), which are likely derived from duplication and diversification of an ancestral ESX-4 system (4). ESX-1 is a key virulence determinant that exports EsxA (ESAT-6) and EsxB (CFP-10) to promote phagosome escape, cytosolic access, and host cell death, whereas ESX-3 is essential for iron and zinc acquisition (5, 6). ESX-2 and ESX-5 are restricted to pathogenic slow-growing mycobacteria.

ESX-5 is the most recently evolved T7SS and the primary pathway for secretion of the PE/PPE protein family, which comprises approximately 7%–10% of the *M. tuberculosis* genome and encodes around 99 PE and 60 PPE proteins characterized by conserved Pro–Glu (PE) and Pro–Pro–Glu (PPE) N-terminal motifs (7). Comparative genomics has demonstrated the expansion of *pe/ppe* genes from nonpathogenic to highly virulent mycobacteria, suggesting that these proteins play critical roles in mediating virulence and host–pathogen interactions (4, 8–10). Structural and biochemical studies have demonstrated stable PE18/PPE26 and PE18/PPE27 heterodimer formation, and these dimers interact with the cytosolic chaperone EspG5 through PPE-specific surface markers. EspG5 maintains the export-competent conformations of ESX-5-specific PPE proteins and delivers heterodimers to the ESX-5 secretion apparatus (11, 12). Because PE/PPE proteins are enriched in T-cell and B-cell epitopes, they are believed to elicit strong immune responses (13, 14). Indeed, PPE26 engages Toll-like receptor 2 (TLR2) to upregulate co-stimulatory molecules (CD80 and CD86) and major histocompatibility complex class I and II on antigen-presenting cells and drive Th1-skewed CD4+ T-cell responses via TLR2/Myd88-dependent signaling (15). However, despite inducing robust host immune responses, ESX-5-­encoded PPE proteins promote mycobacterial survival in macrophages and persistence in mice (11, 15–17), indicating that the elicited immune responses are insufficient for protection against mycobacterial infection (18).

Among the ESX clusters, ESX-5 is unique in that it harbors a tandem *pe/ppe* array (*ppe25–pe18–ppe26–ppe27–pe19*) (19). Phylogenetic analyses indicated that these *pe/ppe* genes co-evolved with ESX-5 following duplication from the ancestral ESX-1 cluster (20), suggesting that ESX-5-encoded PE/PPE proteins play pivotal roles in adaptation to, or counteraction of, the recently evolved host immune system. Deletion of the entire ESX-5 *pe/ppe* substrate set (Mtb∆*ppe25-pe19*) results in marked attenuation in both immunocompromised and immunocompetent mice (21, 22), whereas *Mycobacterium smegmatis* overexpressing *ppe25*, *ppe26*, and *ppe27* exhibits prolonged survival in infected macrophages and mouse tissues (16, 17). These results underscore the importance of ESX-5-encoded PPE proteins in mycobacterial survival. Notably, among 25 *ppe*-overexpressing recombinant *M. smegmatis* strains tested in macrophages, *ppe27* overexpression conferred the greatest enhancement of bacterial survival (23), highlighting its critical role in virulence. According to these studies, PPE27 might play an important role in helping mycobacteria resist macrophage killing or adapt to the intracellular environment, although the underlying mechanism remains unknown.

This study investigated the mechanism by which PPE27 modulates the complex interplay between mycobacteria and the host immune response. To achieve this goal, we generated a *ppe27* deletion mutant of the *Mycobacterium bovis* BCG Tokyo 172 strain (BCG∆*ppe27*) and assessed its impact by profiling host and bacterial transcriptomes simultaneously in macrophages infected with wild-type *M. bovis* BCG Tokyo 172 (wtBCG) or BCG∆*ppe27* using RNA-seq. Our results demonstrated that *ppe27* disruption enhanced host immune responses. In parallel, bacterial RNA (bacRNA)-seq revealed that *JTY_3006* was downregulated in BCG∆*ppe27* relative to its expression in wtBCG during infection. Alignment analysis illustrated that this gene encodes a protein identical to the S-adenosylmethionine (SAM)-dependent methyltransferase (Rv2990c) in *M. tuberculosis* H37Rv (24)*,* which possibly participates in epigenetic regulation in the host and bacteria. Collectively, these findings suggest that PPE27 might actively suppress host inflammation instead of serving solely as an antigenic component, potentially enabling chronic infection and persistence. These results further indicate that *ppe27* deletion could have important implications for future tuberculosis vaccine design.

## RESULTS

### Construction of BCG∆*ppe27* and validation of gene disruption by whole-genome sequencing

Alignment of the 350-amino acid PPE27 sequences of *M. tuberculosis* and *M. bovis* BCG revealed 100% identity (data not shown), indicating functional conservation between these species. The BCG∆*ppe27* mutant was generated from wtBCG by targeted mutagenesis as previously described (25) using the primers listed in Table S1. Previous structural analyzes demonstrated that specific amino acids mediate hydrogen bonding between PE and PPE proteins (26), and *in silico* studies further identified residues required for the interaction of PPE27 with PE18 or EspG5 (11). In our study, the 476-bp deletion disrupted these critical interacting residues (Fig. S1), rendering PPE27 non-functional and incapable of export across the mycobacterial cell envelope. Because PE/PPE family members feature extensive homologous and repetitive sequences that can complicate targeted deletions, whole-genome sequencing was performed on wtBCG and BCGΔ*ppe27* using combined long-read (Oxford Nanopore Technologies, Oxford, UK) and short-read (Illumina, San Diego, CA, USA) platforms. Long- and short-read sequencing confirmed the intended genetic modification at the *ppe27* locus, including the precise 476-bp deletion (Fig. S2A and B). Using the combined long- and short-read approach (Table S2A), only a 1-bp difference in a G homopolymer (37 nt versus 36 nt) was identified in the intergenic region between JTY_RS22225 and JTY_RS20995 (AP010918), in addition to the expected structural differences associated with the construction of the PPE27-deletion mutant; no other variants were identified between the two genomes (Table S2B). Furthermore, reference-genome mapping identified the same set of single-nucleotide polymorphism (SNPs)/insertions and deletions (InDels) in wtBCG and BCG∆*ppe27*, except for this 1-bp intergenic deletion (Table S2C). In addition, sequencing further verified that the single-colony-derived wtBCG and BCGΔ*ppe27* strains were *M. bovis* BCG Tokyo Type II, as previously described (27) (data not shown).

### Parallel host–pathogen RNA-seq and data analysis pipeline

**Fig 1:**
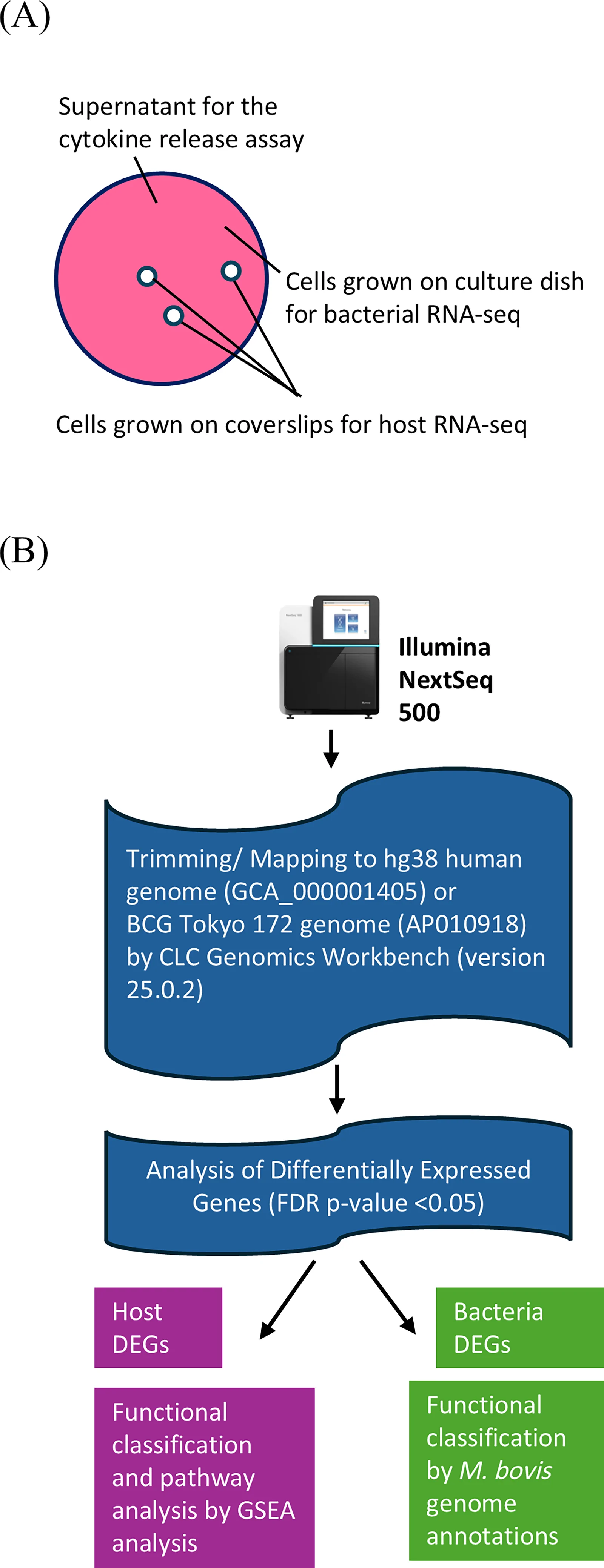
(**A**) Experimental setup of differentiated THP-1 cell infection for parallel RNA-seq. Culture dishes (15 cm) were seeded with 6 × 10^6^ cells, which were infected with each bacterial strain at a multiplicity of infection of 10. Three coverslips were placed in each dish, and the cells grown on the slips were removed upon harvest and lysed for hRNA sequencing. The remaining cells in the culture dish were harvested and disrupted via bead beating for bacRNA sequencing. Supernatants were harvested via filtration through 0.22-μm filters for the cytokine release assay. (**B**) RNA-seq analysis pipeline. RNA-seq data were analyzed using CLCGWB (v25.0.2, Qiagen). Trimmed reads were mapped to human (hg38) or BCG (AP010918) genomes using the RNA-Seq Analysis tool with default parameters. Differential expression analysis was performed using the Differential Expression for RNA-Seq tool. Genes with a false discovery rate (FDR)-adjusted *P*-value < 0.05 and an absolute log2 fold change > 1.5 were considered differentially expressed.

To assess the impact of *ppe27* disruption on host–pathogen interactions, RNA-seq was used to simultaneously profile macrophage and macrophage-associated bacterial transcriptomes during infection. As presented in Fig. 1A, cells grown on coverslips were lysed to extract host RNA (hRNA), whereas the remaining cells in the culture dish were lysed to extract bacRNA. This approach allowed us to obtain sufficient amounts of both host and bacRNA simultaneously for RNA-seq. Culture supernatants from the same dishes were collected for cytokine assays. Three independent biological replicates were processed via infection through library preparation and sequencing. Prior to sequencing, host and bacterial rRNAs were depleted to enrich informative transcripts. FASTQ files were analyzed using CLC Genomics Workbench (CLCGWB version 25.0.2, Qiagen, Hilden, Germany), including quality trimming, alignment to the appropriate reference genomes (Table S3), and identification of differentially expressed genes (DEGs, Fig. 1B).

### DEGs and altered signaling pathways in the host

**Fig 2:**
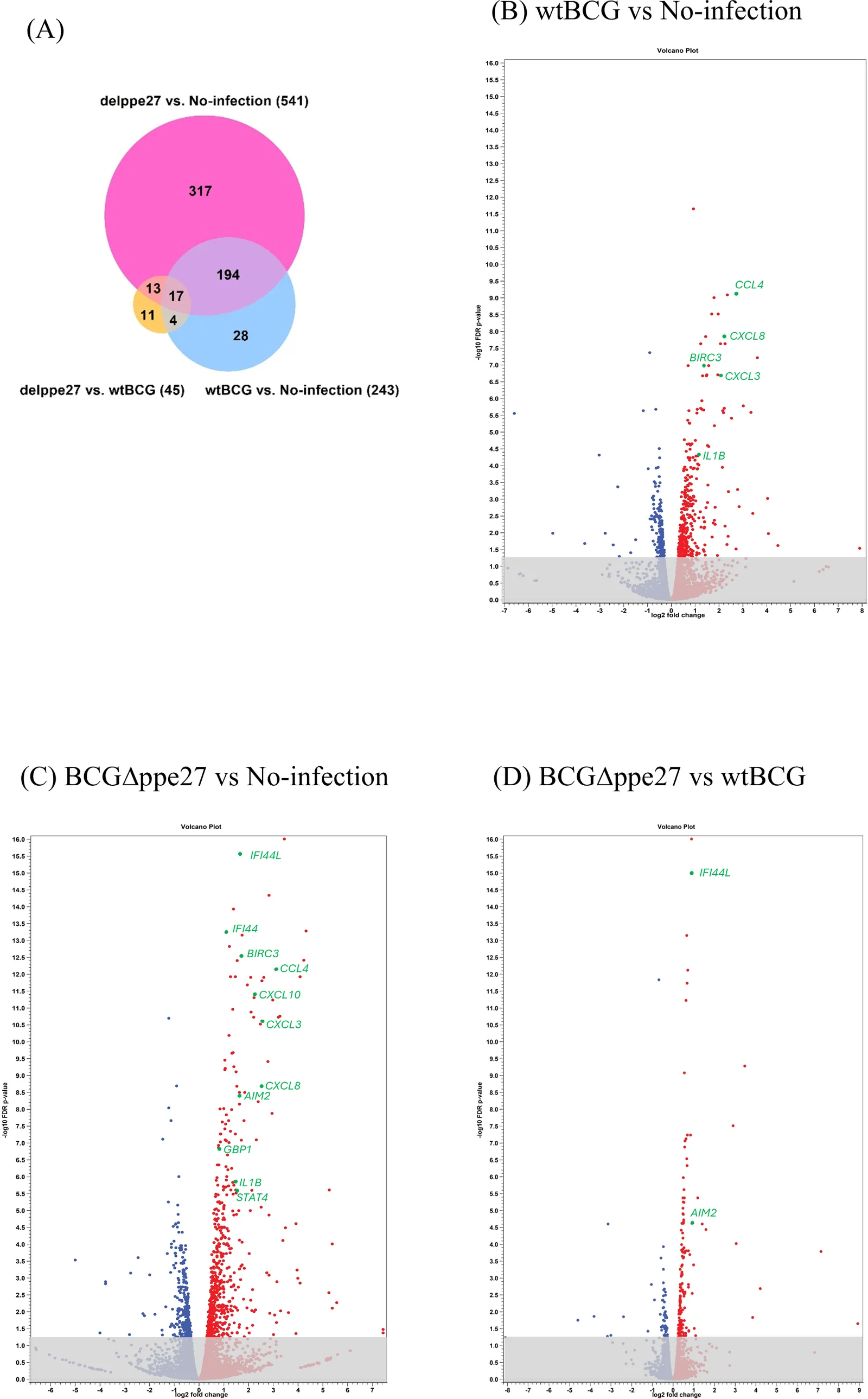
(**A**) Venn diagram illustrating the number of DEGs among wtBCG-infected, BCG∆*ppe27*-infected, and non-infected macrophages (No-infection). (**B–D**) Volcano plots illustrate the up- (red) and downregulated (blue) DEGs. Gray rectangles indicate non-significant DEGs. Positive log2 fold-change values indicate genes upregulated in: (**B**) wtBCG-infected macrophages relative to non-infected cells; (**C**) BCGΔ*ppe27*-infected macrophages relative to non-infected cells; and (**D**) BCGΔ*ppe27*-infected macrophages relative to wtBCG-infected macrophages.

**Fig 3:**
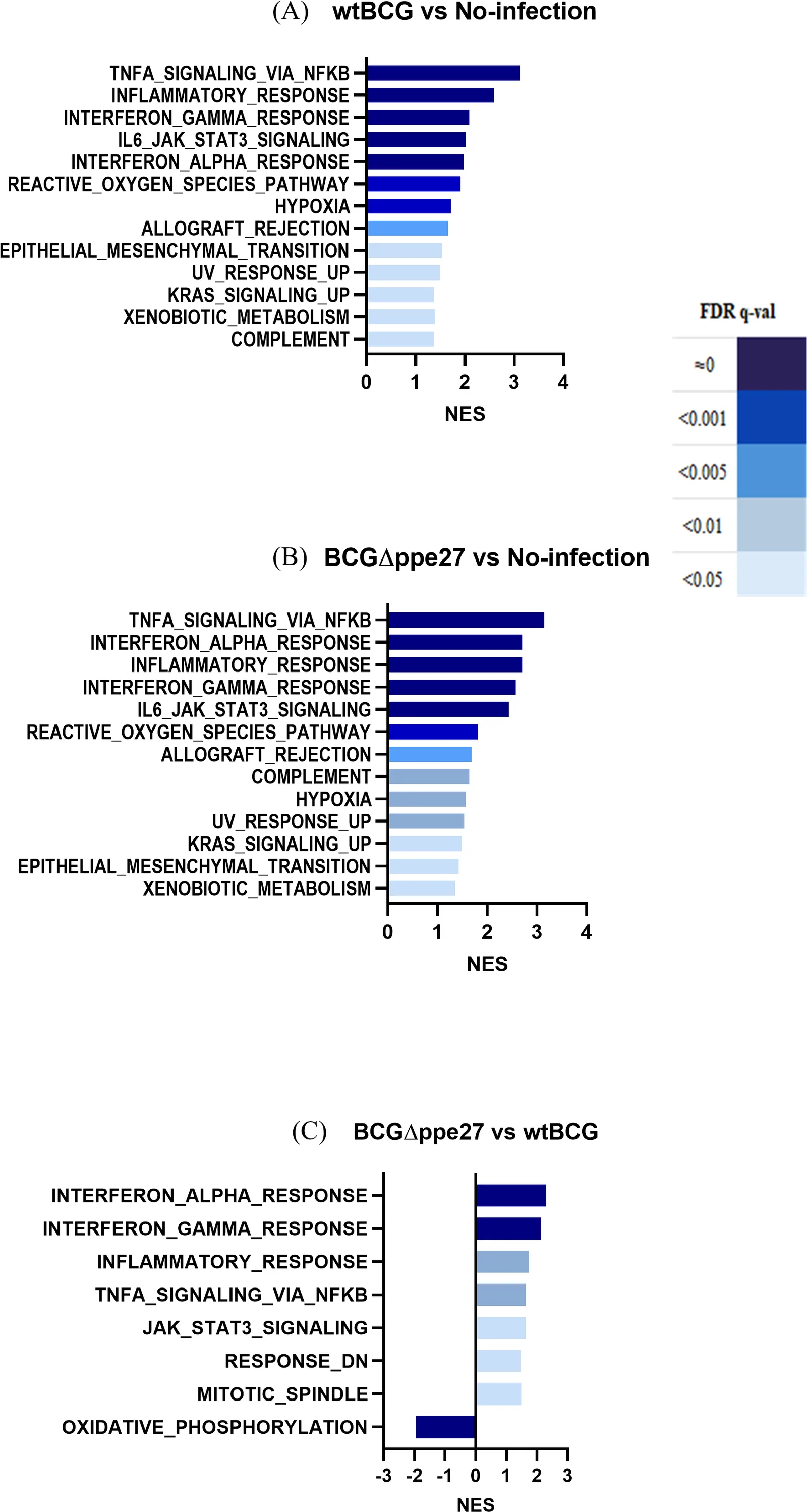
Enriched signaling pathways detected by GSEA. (**A**) Enhanced pathways in wtBCG-infected cells versus non-infected macrophages. (**B**) Enhanced pathways in BCG∆*ppe27*-infected cells versus non-infected macrophages. (**C**) Enhanced pathways in BCG∆*ppe27*-infected cells versus wtBCG-infected cells. NES, normalized enrichment score; FDR < 0.05 was considered statistically significant.

Differential expression analysis using CLCGWB revealed extensive transcriptional reprogramming in macrophages infected with wtBCG or BCG∆*ppe27* compared with the results in the uninfected controls (Fig. 2A; Table S4A and B). wtBCG infection yielded 243 DEGs, whereas BCGΔ*ppe27* infection resulted in 541 DEGs (Fig. 2B and C). By contrast, only 45 DEGs were detected when directly comparing BCG∆*ppe27*- and wtBCG-infected cells (Fig. 2D; Table S4C). Although a significant number of differences were observed in both infected groups relative to uninfected controls, the differential expression between wtBCG and BCG∆*ppe27* was relatively moderate, which, together with batch variation across three independent biological replicates, limited the detection of statistically significant DEGs under the applied threshold (FDR *P*-value < 0.05). To capture coordinated transcriptional changes not evident at the single-gene level, we performed gene set enrichment analysis (GSEA) (28, 29) using the hallmark collection in MSigDB to detect directional enrichment in the predefined signaling pathways. The results revealed significant enrichment of several immune-related pathways, including TNF-α signaling, IFN-α/IFN-γ responses, inflammatory response, and IL-6 signaling, in infected macrophages versus uninfected macrophages (Fig. 3A and B; Table S5A and B). In addition, significant enrichment of IFN-α/IFN-γ responses, inflammatory responses, TNF-α signaling, and IL-6 signaling was successfully identified in BCGΔ*ppe27*-infected cells compared with the findings in wtBCG-infected cells (Fig. 3C; Table S5C), whereas only the oxidative phosphorylation pathway was enriched in wtBCG-infected cells relative to BCGΔ*ppe27*-infected cells (Fig. 3D; Table S5D). This result correlates well with the fact that the upregulation of pro-inflammatory cytokines is promoted by a metabolic shift from oxidative phosphorylation to glycolysis (30).

### Quantitative real-time PCR (RT-qPCR) confirmed the augmented pro-inflammatory responses in BCG∆*ppe27*-infected cells

**Fig 4:**
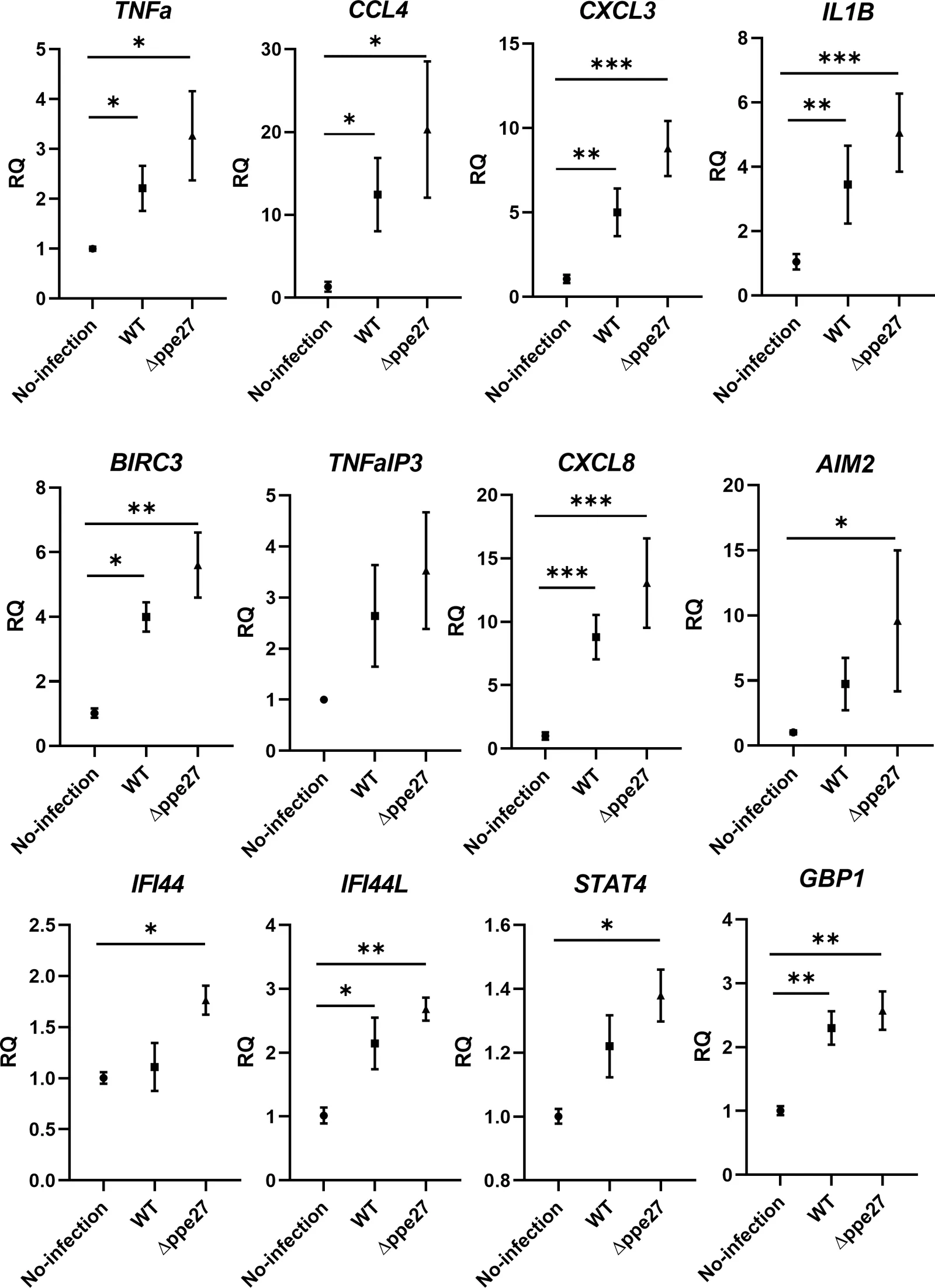
DEGs involved in enhanced signaling pathways were validated by RT-qPCR. Relative gene expression was calculated using the 2^−ΔΔCt^ method, with expression levels normalized to the *β-actin* or *GAPDH* gene, and expressed relative to non-infected cells. Statistical significance was determined using ΔCt values. Data are presented as the mean ± SEM from three biological replicates (*n* = 3). Symbol * indicates *P* < 0.05; ** indicates *P* < 0.01; *** indicates *P* < 0.001. WT, wild-type.

*M. bovis* BCG vaccination in healthy humans induces pro-inflammatory cytokines such as IL-1β, TNF-α, and IL-6 (13, 14). Consistently, our GSEA results revealed marked enhancement of the TNF-α pathway in the infected groups. To further validate this observation, the expression of *TNF* and its downstream gene set, including *CCL4, CXCL3, IL1B,* and *BIRC3,* were quantified by RT-qPCR (Fig. 4). The results demonstrated significant upregulation of *TNF*, *CCL4, CXCL3, IL1B,* and *BIRC3* in both wtBCG- and BCG∆*ppe27*-infected cells compared with their expression in uninfected controls. The expression of *TNFAIP3*, a negative regulator of *TNF* and *IL1B* signaling, was non-significantly increased in both infection groups. In addition to pro-inflammatory cytokine responses, mycobacterial infection robustly stimulates the production of chemokines, such as CXCL8, a molecule known to limit mycobacterial survival within macrophages. RT-qPCR confirmed the significant elevation of *CXCL8* transcripts in both infection groups. Furthermore, RNA-seq analysis revealed marked upregulation of the double-stranded DNA sensor *AIM2* in both infection groups (Table S4A and B), and we confirmed that its expression was significantly higher in BCG∆*ppe27*-infected cells by RT-qPCR (Fig. 4). In addition, the upregulation of several genes involved in IFN-α and IFN-γ signaling pathways was confirmed. *IFI44* and *STAT4* were significantly upregulated in BCG∆*ppe27*-infected cells compared with non-infected controls, whereas *IFI44L* and *GBP1* were upregulated in both wt- and BCG∆*ppe27*-infected cells relative to non-infected controls (Fig. 4).

### Enhanced cytokine production in BCGΔ*ppe27*-infected cells

**Fig 5:**
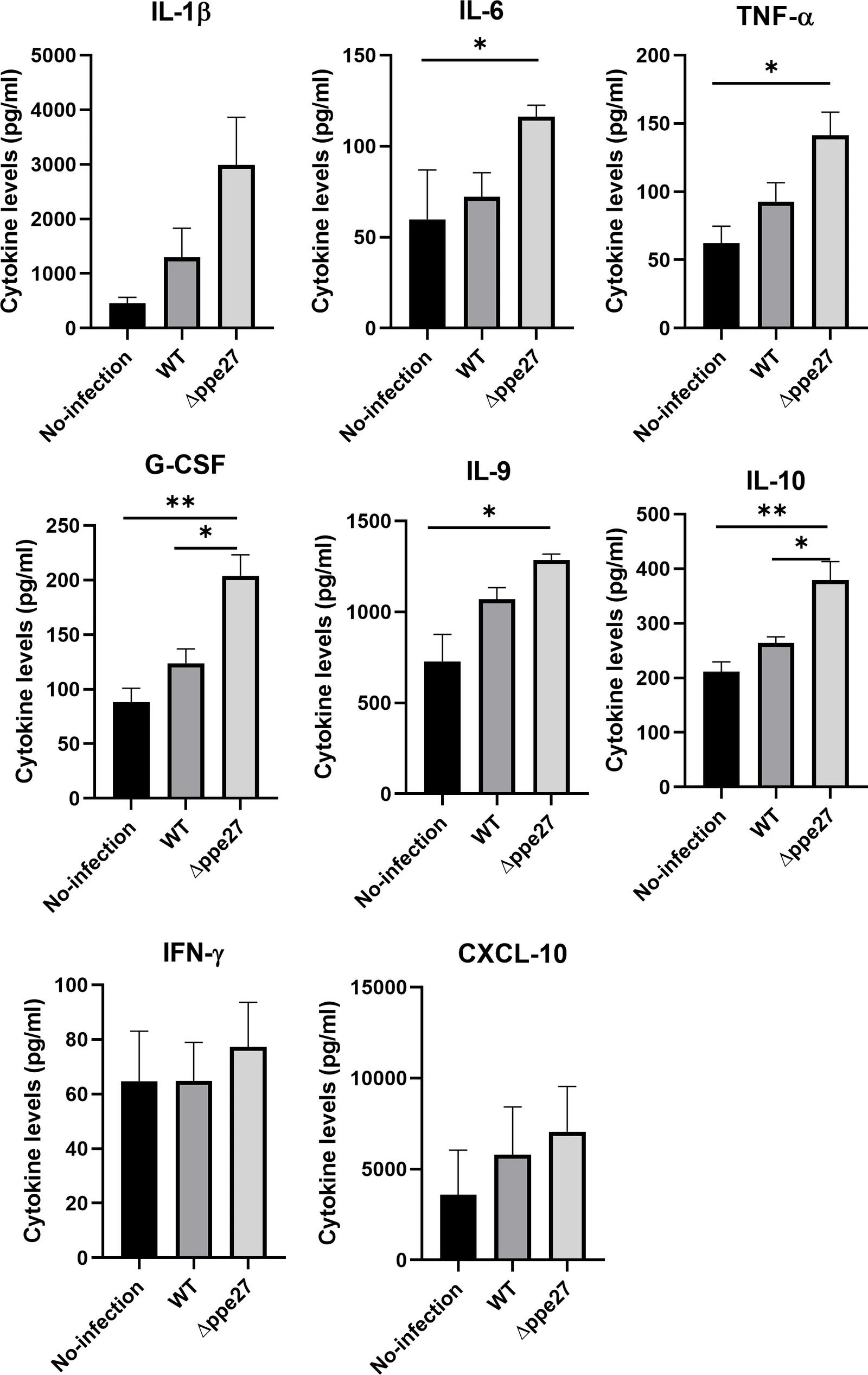
Measurement of secreted cytokines in the culture supernatants of wtBCG-infected, BCG∆*ppe27*-infected, and non-infected macrophages using a 27-plex cytokine assay. Data are presented as mean ± SEM from three biological replicates (*n* = 3). Symbol * indicates *P* < 0.05; ** indicates *P* < 0.01. G-CSF, granulocyte colony-stimulating factor; WT, wild-type.

The results of GSEA and RT-qPCR demonstrated elevated immune responses in BCG∆*ppe27*-infected macrophages compared with those in wtBCG-infected or uninfected cells. To assess whether these transcriptional changes were reflected at the protein level, culture supernatants were collected and analyzed for 27 cytokines using a multiplex cytokine assay. The results illustrated that G-CSF and IL-10 expression was significantly elevated in BCG∆*ppe27*-infected cells versus wtBCG-infected or uninfected cells (Fig. 5). TNF-α, IL-6, and IL-9 expression levels were significantly elevated in BCGΔ*ppe27*-infected cells compared with those in uninfected control cells and tended to be higher than those in wtBCG-infected cells, although the differences between the two infected groups did not reach statistical significance. A similar trend was also observed for IL-1β, IFN-γ, and CXCL-10. These findings suggest that deletion of *ppe27* enhances host inflammatory responses, at least in part at the protein level.

### RNA-seq of bacterial transcriptomes under broth-grown and macrophage-associated conditions

**Fig 6:**
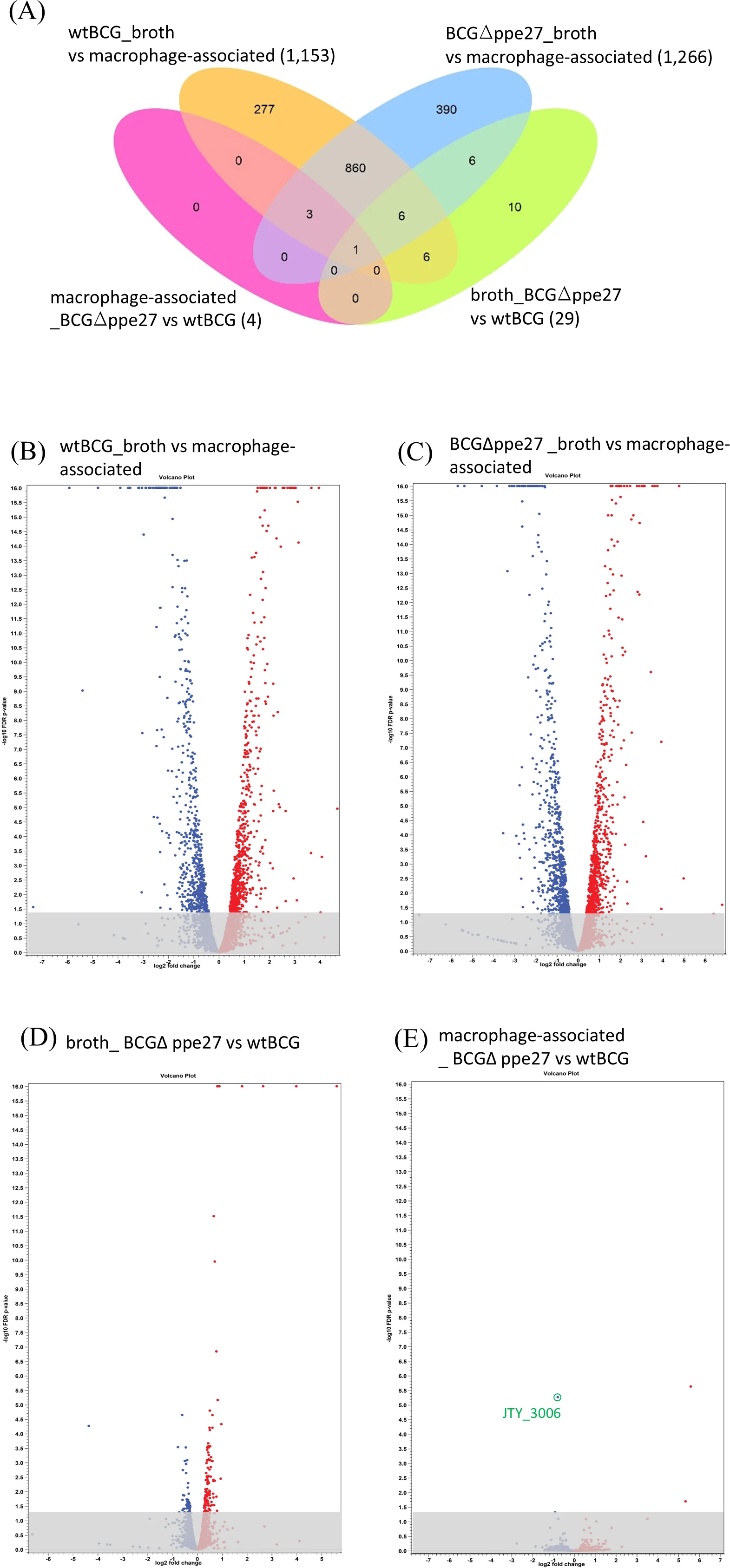
(**A**) Venn diagram of bacterial DEGs. Comparison of broth-grown and macrophage-associated RNA transcripts identified 1,153 DEGs in wtBCG and 1,266 DEGs in BCG∆*ppe27*. In parallel, comparison of RNA transcripts between two strains identified 29 DEGs in broth-grown condition and 4 DEGs in macrophage-associated condition. (**B–E**) Volcano plots illustrate upregulated (red) and downregulated (blue) DEGs. Gray rectangles indicate the non-significant region. Positive log2 fold-change values indicate genes upregulated in: (**B**) wtBCG grown in broth relative to wtBCG recovered from infected macrophages; (**C**) BCGΔ*ppe27* grown in broth relative to BCGΔ*ppe27* recovered from infected macrophages; (**D**) the BCGΔ*ppe27* group relative to the wtBCG group in bacterial broth culture; and (**E**) the BCGΔ*ppe27* group relative to the wtBCG group in infected macrophages. DEGs were defined using the following significance criteria: FDR < 0.05 and absolute fold change > 1.5.

**Fig 7:**
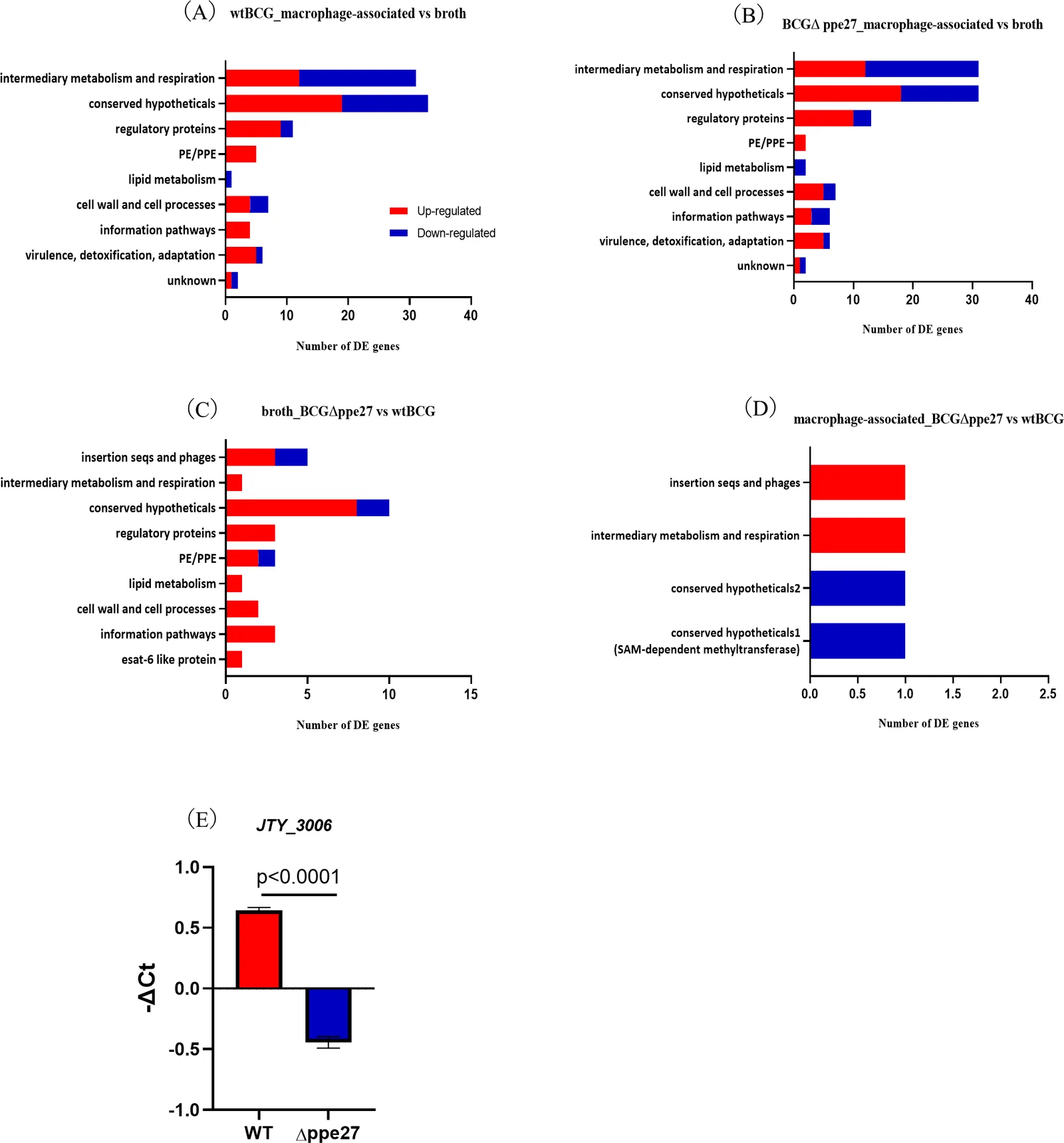
Bacterial DEGs (top 100) in macrophage-associated versus broth-grown conditions and functional annotations in wtBCG (**A**) and BCG∆*ppe27* (**B**). The comparison of BCG∆*ppe27* to wtBCG revealed 29 DEGs in broth-grown (**C**) and 4 DEGs in macrophage-associated (**D**) conditions. (**E**) RT-qPCR confirms downregulation of the SAM-dependent methyltransferase gene *JTY_3006* in BCGΔ*ppe27* compared with wtBCG.

Parallel RNA-seq was performed to characterize bacterial transcriptomic changes under macrophage-associated conditions. Because the impact of *ppe27* disruption on the bacterial transcriptome under broth-grown conditions has not been previously examined, bacteria grown in 7H9 broth were also harvested at log-phase, and bacRNA was isolated for broth-grown RNA-seq. The transcriptomic profiles of wtBCG and BCG∆*ppe27* were then compared under both broth-grown and macrophage-associated conditions by analyzing DEGs. This analysis revealed pronounced transcriptional reprogramming when comparing broth-grown and macrophage-associated conditions in both wtBCG (1,153 DEGs) and BCG∆*ppe27* (1,266 DEGs), confirming that host–pathogen interactions trigger extensive changes in bacterial gene expression (Fig. 6A; Tables S6A and S7A). Direct comparison between strains identified 29 DEGs in BCG∆*ppe27* relative to wtBCG under broth-grown conditions, whereas only four DEGs were detected under macrophage-associated conditions (Fig. 6A; Table S8 and S9). Notably, the overall patterns of gene upregulation and downregulation were comparable between the two strains under both broth-grown and macrophage-associated conditions, as illustrated in the volcano plots (Fig. 6B and 7C). Under broth-grown conditions, *ppe27* deletion led to slightly altered gene expression in BCG∆*ppe27* versus wtBCG (Fig. 6D), whereas only a limited impact was observed under macrophage-associated conditions (Fig. 6E).

### Functional annotation of DEGs in bacterial cells

As we observed substantial changes in transcriptomes when comparing broth-grown and macrophage-associated conditions in both bacterial strains, we next examined which biological functions were involved and influenced by *ppe27* disruption by exploiting functional characterization. This was achieved by annotating the most significant top 100 DEGs to the functional categories, as described in the Materials and Methods. The bacterial expression profiles under broth-grown and macrophage-associated conditions exhibited similar expression patterns between wtBCG and BCG∆*ppe27* (Fig. 7A and B; Table S6B and Table S7B). Notably, genes encoding regulatory proteins, PE/PPE proteins, and proteins involved in virulence, detoxification, and adaptation were upregulated under macrophage-associated conditions versus broth-grown conditions in both strains, indicating that host entry triggers the expression of genes associated with counteracting host immune defenses. Interestingly, under broth-grown conditions, 24 of the 29 identified DEGs involved in metabolism or cell process were upregulated in BCG∆*ppe27* compared with their expression in wtBCG (Fig. 7C; Table S8), indicating that BCG∆*ppe27* is more physiologically active. However, when comparing the transcriptomes of the two strains under macrophage-associated conditions, only four DEGs were identified (Fig. 6E and 7D; Table S9), suggesting that *ppe27* loss has a limited impact on bacterial transcription itself. Closer inspection of *JTY_3135* and *sdhD_3* revealed that sequencing reads corresponding to these genes mapped to multiple genomic locations. *JTY_3135* is an IS*1081* transposase sequence, and BCG Tokyo 172 has five copies on its genome. BCG Tokyo 172 has triple tandem duplications of the genomic region, including the genes from *astB* to *sdhD*, and there are three identical *sdhD3* sequences (31). Given the abundance of repetitive sequences in the BCG Tokyo 172 genome, such multi-mapping is likely to introduce mapping-related quantification bias, particularly in short-read sequencing approaches. In contrast, sequencing reads mapped to *JTY_0110* and *JTY_3006* were confirmed to align uniquely to their respective genomic loci. RT-qPCR further validated the downregulation of *JTY_3006* in BCGΔ*ppe27* compared with wtBCG (Fig. 7E). In contrast, *JTY_0110* expression was detected only marginally above the RT-PCR detection threshold (Fig. S4), precluding a reliable assessment of its differential expression. *JTY_3006* was previously annotated as a conserved hypothetical protein (32); however, our BLAST analysis identified it as a SAM-dependent methyltransferase, as its amino acid sequence shares 100% identity with that of Rv2990c in *M. tuberculosis* H37Rv (24).

To determine whether reduced expression of *JTY_3006* could influence the bacterial burden in macrophages, CFU assays were performed. The result showed that the macrophage-associated bacterial burdens of BCGΔ*ppe27* and wtBCG were comparable at 24 h post-infection (Fig. S5), although BCGΔ*ppe27* was more efficiently associated with or internalized by macrophages at 4 h post-infection (Fig. S6).

## DISCUSSION

In the present study, parallel host–pathogen RNA-seq analysis demonstrated that *ppe27* disruption significantly enhanced host pro-inflammatory responses, including the TNF-α, IFN, and IL-6 pathways, despite only moderate transcriptomic shifts in the bacteria themselves. The concurrent downregulation of a SAM-dependent methyltransferase in the BCGΔ*ppe27* mutant implies a potential epigenetic link for modulating host responses, although its underlying mechanism remains to be defined.

TNF-α is a central mediator of host defense against tuberculosis, contributing to phagosome maturation, antigen presentation, and granuloma formation, and its inhibition is associated with an increased risk of disease reactivation (33–36). Avirulent mycobacterial strains have been reported to elicit higher levels of TNF-α from infected macrophages than *M. tuberculosis*, suggesting that attenuation of the TNF-α response is important for virulence (37). Consistent with this, *M. tuberculosis* has been demonstrated to modulate host immune responses, including TNF-α production, and thereby support bacterial survival and pathogenesis (38, 39). In the present study, GSEA analysis suggested that *ppe27* disruption was associated with increased TNF-α signaling. In addition, upregulated expression of the anti-apoptotic gene, *BIRC3* (also known as *cIAP2*), might contribute to prolonged macrophage survival and sustain pro-inflammatory responses in BCGΔ*ppe27*-infected macrophages. Notably, BIRC3 has also been reported to influence macrophage differentiation and drive polarization toward the pro-inflammatory M1 phenotype, a process characterized by the upregulation of *TNF* (40). Furthermore, concurrent elevation of TNF-α and IL-10 was observed in BCGΔ*ppe27*-infected macrophages (Fig. 5). IL-10 is a potent anti-inflammatory cytokine whose elevation might reflect a compensatory response to increased pro-inflammatory cytokine production. TNF-ɑ and IL-10 are often induced simultaneously during mycobacterial infection, and their balance might influence macrophage fate toward apoptosis or survival (41). These findings highlight a strong correlation between PPE27 and the modulation of host TNF-α signaling.

We also demonstrated that several components of the IFN signaling pathway, including *STAT4*, *IFI44*, *IFI44L*, *GBP-1, AIM2, and IL1B*, were either significantly and uniquely upregulated in BCGΔ*ppe27*-infected macrophages or exhibited higher expression levels compared with those in wtBCG-infected macrophages. Notably, *IFI44* and *IFI44L*, accompanied by pro-inflammatory cytokine and chemokine release, have been reported to enhance *M. tuberculosis* clearance in human macrophages (42, 42, 43). *GBP-1* is associated with the AIM2 inflammasome pathway, a cytosolic pattern recognition system that detects extracellular DNA and promotes downstream IL-1β secretion, thereby contributing to protection against *M. tuberculosis* infection (44, 45). Similarly, ESX-1-proficient recombinant BCG vaccine candidates have been shown to induce greater IL-1β release, which correlates with higher protective efficacy (46).

Collectively, our results imply that PPE27 might dampen inflammatory/IFN signaling during primary infection, whereas *ppe27* overexpression in a trained-immunity vaccination context has been reported to enhance glycolysis-linked responses (47). In addition, infection by *ppe27*-overexpressing *M. smegmatis* activates TNF-α signaling and suppresses IL-6 in ANA-1 macrophages (17). One possible explanation for the discrepancies in response to overexpression and deletion mutants could be that other PPE proteins play overlapping or compensatory roles, which complicates the responses in different experimental contexts (22, 45). We cannot exclude the possibility that deletion of *ppe27* may have indirect effects on the secretion of other ESX-5 substrates, which represents one limitation of this study. Furthermore, because the bacterial burden was higher at 4 h post infection (Fig. S6), suggesting that BCGΔ*ppe27* was more efficiently associated with or internalized by macrophages. Therefore, the differences observed in the host transcriptome at 24 h post infection may have been influenced by differences in early bacterial exposure, bacteria-per-cell distribution, or attachment/internalization dynamics. Accordingly, under the conditions examined in this study, the macrophage responses associated with BCGΔ*ppe27* infection cannot be attributed solely to the loss of PPE27 and should be interpreted in the context of these potential confounding factors.

The results of bacRNA-seq demonstrated that bacteria actively remodel their transcriptomes during host–bacteria interactions (Fig. 7A and B), suggesting evolved mechanisms that promote bacterial survival within the host. Although more than 1,000 DEGs were identified when comparing gene expression between broth-grown and macrophage-associated conditions, functional analysis of the top 100 DEGs revealed broadly similar patterns of up- and downregulation between wtBCG and BCGΔ*ppe27*, suggesting that *ppe27* disruption does not grossly alter the overall bacterial transcriptional response to the host environment. Notably, genes encoding regulatory proteins, PE/PPE proteins, and proteins involved in virulence, detoxification, and adaptation were consistently upregulated upon the transition from broth-grown to macrophage-associated conditions, reflecting bacterial responses to counteract host defenses for survival (36). Bacterial lipid metabolism was suppressed upon entering the host (Fig. 7A and B), consistent with previous observations that mycobacteria can utilize host-derived lipids to reduce the demand on their own lipid metabolic pathways (48).

The mycobacterial protein PPE27 has been reported to be inducible upon infection (49), consistent with our bacRNA-seq result highlighting upregulation of *ppe27* in wtBCG-infected cells compared with that under the broth condition (Table S6A), indicating that PPE27 plays a critical role in bacterial pathogenesis. When comparing DEGs between wtBCG and BCGΔ*ppe27* under broth conditions, 24 of the 29 identified DEGs were upregulated in BCGΔ*ppe27*, whereas only a limited number of DEGs were detected under macrophage-associated conditions, indicating that transcriptional differences are largely constrained in the macrophage-associated condition. Among the limited number of DEGs, we identified a class I SAM-dependent methyltransferase (JTY_3006) that was downregulated in BCGΔ*ppe27*. Methyltransferases participate in diverse cellular processes, including metabolism, gene regulation, and host–pathogen interactions. Several mycobacterial methyltransferases are implicated in virulence through modulation of host immune responses via epigenetic mechanisms. For example, the secreted methyltransferase Rv1988 localizes to the host nucleus and methylates histone H3 at arginine 42, thereby repressing genes involved in early antimicrobial defense (50). Similarly, Rv2067c trimethylates histone H3K79 in macrophages, facilitating bacterial resistance to host inflammatory and oxidative responses (51). A recent study revealed that a SAM-dependent methyltransferase mediates the methylation of the glycoside moiety of mycofactocin in *M. smegmatis,* although its contribution to bacterial growth remains unclear (52). In this study, we identified *JTY_3006* as a candidate DEG, although there is no direct evidence currently to connect its relationship to PPE27, host epigenetic modifications, or the observed inflammatory phenotype.

We acknowledge the absence of a complementation study as a weakness of this work; consequently, a causal relationship between PPE27 and the observed phenotypes cannot be established, although our whole genome sequencing analyzes identified no additional intragenic or structural variants beyond the intended genetic modifications at the *ppe27* locus. Taken together, our findings support the idea that some PPE proteins help mycobacteria control host inflammation and that manipulating even a single bacterial gene can reshape both pathogenicity and vaccine efficacy.

## MATERIALS AND METHODS

### Bacterial strains and culture conditions

The *M. bovis* BCG Tokyo 172 vaccine strain (GenBank: AP010918) was obtained from the Japan BCG Laboratory (Tokyo, Japan). Three milliliters of bacteria were inoculated and cultured in 30 mL Middlebrook 7H9 medium (Becton, Dickinson and Company, USA) using a T75 flask supplemented with 0.05% Tween 80, 0.2% glycerol, and 10% albumin–dextrose–catalase (Becton, Dickinson and Company) without shaking, or on Middlebrook 7H10 agar containing 0.2% glycerol and 10% oleic acid–albumin–dextrose–catalase (Becton, Dickinson and Company) and incubated for 3 wk. When required, kanamycin (25 µg/mL) and hygromycin (50 µg/mL) were added to the media. Bacteria cultures were incubated at 37°C, harvested in the mid-exponential phase, and passed through a 5-μm pore size filter (Pall Corporation) to obtain a single-cell suspension prior to infection (53).

### Generation of the BCG∆*ppe27* deletion mutant and whole-genome sequencing

BCG∆*ppe27* was generated by allelic exchange as previously described (25, 54). Briefly, genomic DNA from wtBCG was extracted using the ISOPLANT II Kit (Nippon Gene, Tokyo, Japan) and used as a template for PCR amplification. A fragment encompassing *ppe27* was amplified using primers ppe27Del-F and ppe27Del-R (Table S1) and subcloned into the EcoRV-linearized pGEM vector. In a subsequent round of PCR, the primers ppe27-Ins-F and ppe27-Ins-R were used to introduce a hygromycin B resistance cassette flanked by upstream and downstream sequences of the target gene, with the subcloned construct serving as a template. The hygromycin resistance cassette was fused to the amplified fragments via In-Fusion cloning (Takara Bio, Shiga, Japan). The resulting constructs, containing the hygromycin cassette and flanking partial target gene sequences, were electroporated into wtBCG cells to generate the mutant BCG∆*ppe27*. Homologous recombination of the target gene *ppe27* was facilitated by co-expression of the mycobacteriophage proteins Che9c gp60 and gp61, which were derived from the pJV53 plasmid (https://www.addgene.org/26904/). Additionally, wtBCG cells were electroporated with the empty pJV53 plasmid to control for background effects. The electroporated cells were recovered overnight at 37°C before being spread onto 7H10 plates containing 50 mg/L kanamycin and 50 mg/L hygromycin, followed by incubation for 3 wk. Colonies carrying the *ppe27* deletion were confirmed by PCR and propagated in 7H9 medium to the mid-exponential phase. The bacterial cultures were then passed through a 5-μm syringe filter to obtain a single-cell suspension and stored at −80°C until use. To confirm successful *ppe27* deletion without off-target mutations, genomic DNA from wtBCG and BCG∆*ppe27* was isolated and subjected to long-read genome sequencing using MinION (R10.4.1, Oxford Nanopore Technologies) and short-read genome sequencing using the Miseq Reagent kit v2 (500 cycles, Illumina). The sequencing statistics were listed in Table S2A. Nanopore reads longer than 6,000 bp were used for *de novo* genome assembly with Flye v2.9.6. The resulting contigs were refined by incorporating sequence information from local assemblies of MiSeq short reads. Sequence differences between the wild-type and PPE27-deleted BCG strains were assessed by whole-genome alignment using MUMmer v3.23 (Table S2B). MiSeq short reads were mapped to the BCG Tokyo reference genome (AP010918), and SNPs and InDels were subsequently identified using CLCGWB under the conditions described in Table S2A through C.

### Culture of THP-1 cells and infection

Human THP-1 cells were obtained from RIKEN BRC (Japan) and cultured in RPMI-1640 medium supplemented with 10% fetal bovine serum, 2 mM l-glutamine, 100 U/mL penicillin–streptomycin, and sodium bicarbonate. Cells were maintained at 37°C in a humidified atmosphere of 5% CO_2_. For the parallel host–pathogen RNA-seq experiment, 6 × 10^6^ THP-1 cells were seeded in a 15-cm untreated culture dish containing three sterile 18-mm cover glasses. THP-1 cells were differentiated via treatment with 10 ng/mL phorbol 12-myristate 13-acetate for 24 h, followed by washing with fresh medium and 48 h of additional incubation. Prior to BCG infection, cells were washed three times with PBS, maintained in antibiotic-free medium, and infected with wtBCG (6.3 × 10^7^ CFU/mL) or BCG∆*ppe27* (5.3 × 10^7^ CFU/mL) at a multiplicity of infection (MOI) of 10.

### Host and bacRNA isolation

Three biological replicates were included in this study. A biological replicate was defined as an independent experiment encompassing infection, RNA isolation, library preparation, and RNA sequencing. This workflow—from infection through sequencing—was repeated to generate second and then third replicates performed on different days. Thus, all three replicates were derived from fully independent experiments. At 24 h post-infection, the supernatant was collected and filtrated through a 0.22-μm filter (MilliporeSigma, Burlington, MA, USA) to remove all cellular debris. The filtrated supernatant, containing secreted cytokines, was then stored at −80°C until use. The cells were then washed three times with PBS and incubated for 5 min between each wash to remove non-attached or non-internalized bacilli. Three cover glasses containing infected cells were transferred to a new culture dish containing TRIzol (Thermo Fisher Scientific, Waltham, MA, USA) and suspended to lyse the cells for subsequent hRNA extraction. The remaining cells in the 15-mL culture dish were lysed and scraped with 1% SDS to maximize the quantity of bacRNA for RNA-seq. Bacteria associated with the host cells were concentrated via centrifugation at 15,000 × *g* at 4°C for 15 min, and the bacterial pellets were suspended in 1 mL TRIzol and transferred to a 2-mL tube containing Lysing Matrix B before disrupting the bacteria by FastPrep-24 (MP Biomedicals). Host and bacterial RNAs were isolated separately and purified using RNeasy Mini Kit (Qiagen) with on-column DNase treatment.

### Parallel host–pathogen RNA-seq

Excessive rRNA in RNA samples can significantly reduce the sensitivity of mRNA detection. To avoid this, rRNA was depleted prior to library preparation using the Ribo-Zero rRNA Removal Kit (human/mouse/rat) (Illumina) for hRNA. For bacRNA samples, we depleted rRNA using the Ribo-Zero rRNA Removal Kit (human/mouse/rat) in combination with the Ribo-Zero rRNA Removal Kit (gram-positive bacteria) (Illumina). hRNA libraries were constructed using the TrueSeq Stranded mRNA Sample Preparation Kit (Illumina) with fragmentation at 94°C for 8 min. bacRNA libraries were prepared using the NEBNext Ultra II Directional RNA Library Prep Kit (New England Biolabs, Ipswich, MA, USA) without a fragmentation step. cDNA libraries were quantified using a Qubit 2.0 fluorometer (Thermo Fisher Scientific), and the size distribution was assessed using either the DNA-1000 kit (for hRNA libraries) or the DNA High Sensitivity kit (for bacRNA libraries) on an Agilent 2100 bioanalyzer (Agilent, Santa Clara, CA, USA). Libraries were normalized to equal concentrations and pooled as three-plex for host (hRNA libraries: uninfected control, wtBCG-infected, and BCG∆*ppe27*-infected groups) or two-plex for bacteria (bacRNA libraries: wtBCG and BCG∆*ppe27*) prior to sequencing. Sequencing was conducted on an Illumina NextSeq 500 platform using the NextSeq 500/550 Mid Output v2 kit (2 × 76 cycles) for RNA-seq. For RNA-seq using broth-grown bacteria, sequencing was conducted using an Illumina MiSeq using Reagent Kit v3 (150 cycles).

### Bioinformatics analysis for RNA-seq

RNA-seq FASTQ files obtained from sequencing were processed using CLCGWB (v25.0.2) to trim the adaptor sequences and map the trimmed reads to the reference genomes using GRCh38 (hg38) (GCA_000001405) for human genes and BCG Tokyo 172 (GenBank no. AP010918) for bacterial genes. DEGs were identified using the “Differential Expression for RNA-Seq” tool in CLCGWB, which uses a generalized linear model (GLM) assuming that read counts follow a negative binomial distribution. Trimmed mean of M-values normalization was applied to account for differences in library composition across samples. Each infection-day batch included samples from all experimental groups, and infection day was included as a covariate in the analysis to account for batch effects. To assay macrophage-associated bacterial DEGs, the trimmed reads were first mapped to the human genome, and the unmapped reads were extracted and mapped to the bacterial genome to remove human genome contamination. The significance criteria were set to FDR < 0.05 and |fold change| > 1.5.

### GSEA

The GSEA v4.4.0 (https://www.gsea-msigdb.org/gsea/index.jsp) tool was employed to analyze functionally enriched pathways across three groups of cells (uninfected, wtBCG-infected, and BCG∆*ppe27*-infected), identifying significantly enriched pathways. Hallmark gene sets (v2025.1.Hs.symbols) were chosen as the gene sets database, and the chip platform was set to ENSEMBL_Gene_ID_MSigDB v2025.1Hs. The analysis used 1,000 permutations with the permutation type as “gene set.” A *t*-test was used for all enrichment calculations and metrics for ranking genes. FDR *q*-value <0.05 was defined as the threshold for statistical significance.

### RT-qPCR

RT-qPCR was performed to validate mRNA expression determined using RNA-seq data. In total, 100 ng of hRNA were reverse-transcribed into cDNA using SuperScript III Reverse Transcriptase (Thermo Fisher Scientific). RT-qPCR was conducted using the Power SYBR Green PCR Master Mix (Thermo Fisher Scientific). Primer sequences for the detection of host genes, including *IL1B, TNF, CXCL3, BIRC3, IL6, IL10, IL9*, and *GCSF*, as well as the bacterial gene *JTY_3006*, were designed using Primer3Plus (https://www.primer3plus.com/index.html) and are listed in Table S1. Detection of *IFI44, IFI44L, STAT4*, and *GBP1* gene expression was performed using TaqMan probe assays, and the corresponding assay IDs are listed in Table S1. The 2^−ΔΔCt^ method was used to quantify and compare gene expression normalized to the housekeeping gene *β-actin* or *GAPDH* as described elsewhere (55). Statistical significance testing was performed on ΔCt values, and expression fold changes were shown as 2^−ΔΔCt^.

### Multiplex cytokine assay

At 24 h post-infection, the supernatant of THP-1 cells infected with wtBCG or BCG∆*ppe27* was collected and filtrated through a 0.22-μm filter (Millipore) to remove any cellular debris. The filtered supernatants were stored at −80°C until analysis. The concentration of cytokines released during infection was quantitatively measured using the Bio-Plex 200 system with the Bio-Plex Pro Human Cytokine 27-Plex Immunoassay (Bio-Rad, Hercules, CA, USA), following the manufacturer’s protocol. Data acquisition was performed using Bio-Plex Manager software (Bio-Rad) on the Bio-Plex 200 instrument.

### Functional annotation of bacterial DEGs

The *M. bovis* BCG Tokyo 172 genome annotation data have been previously published (32). We annotated DEG products and assigned functional categories using an annotation file downloaded from the NCBI database (https://www.ncbi.nlm.nih.gov/datasets/gene/ GCA_000010685.1/) and data from Mycobrowser, which utilizes the *M. bovis* genome AF2122/97 (https://mycobrowser.epfl.ch/releases). Based on the results of this functional analysis, the top 100 significant genes in wtBCG and BCG∆*ppe27* under broth-grown and macrophage-associated conditions were plotted (Fig. 7A and B). The significance criteria were set to FDR < 0.05 and |fold change| > 1.5.

### Statistical analysis

Statistical analyses were performed using GraphPad Prism V8.4 software (GraphPad Software, Inc., Boston, MA, USA). Groups were analyzed for significant findings using one-way analysis of variance tests followed by Tukey’s test for multiple comparisons. All data are presented as the mean ± SEM, and *P* < 0.05 was considered statistically significant.

## Supplementary Material

Reviewer comments

## Data Availability

Raw sequence data have been deposited in the DRA database under the accession numbers DRA029372–DRA029374, and DRA029729.

## SUPPLEMENTAL MATERIAL

Supplemental figures spectrum.00915-26-s0001.pdf Fig. S1 to S6.

Table S1 spectrum.00915-26-s0002.xlsx List of primer sequences.

Table S2 spectrum.00915-26-s0003.xlsx Whole-genome sequencing.

Table S3 spectrum.00915-26-s0004.xlsx RNAseq metrics.

Table S4 spectrum.00915-26-s0005.xlsx Host DEGs list.

Table S5 spectrum.00915-26-s0006.xlsx GSEA_Hallmark.

Table S6 spectrum.00915-26-s0007.xlsx Bacteria DEGs_wtBCG_broth vs. macrophage-associated.

Table S7 spectrum.00915-26-s0008.xlsx Bacteral DEGs_delppe27_broth vs. macrophage-associated.

Table S8 spectrum.00915-26-s0009.xlsx Bacterial DEGs_broth_delppe27 vs. wtBCG.

Table S9 spectrum.00915-26-s0010.xlsx Bacterial DEGs_macrophage-associated_delppe27 vs. wtBCG.

OPEN PEER REVIEW reviewer-comments.pdf An accounting of the reviewer comments and feedback.

ASM does not own the copyrights to Supplemental Material that may be linked to, or accessed through, an article. The authors have granted ASM a non-exclusive, world-wide license to publish the Supplemental Material files. Please contact the corresponding author directly for reuse.
