# Supplementary material for: Deletion of ppe27 in Mycobacterium bovis BCG augments macrophage immune responses during infection
Source: Microbiol Spectr. 2026 Oct 6;14(11):e00915-26. doi: 10.1128/spectrum.00915-26 (PMC13729191; doi:10.1128/spectrum.00915-26)
Supplement: Supplementary file 1 [file reviewer-comments.pdf]

ASM | Microbiology Spectrum

Deletion of *ppe27* in *Mycobacterium bovis* BCG augments macrophage immune responses during infection

TZ-Chun Guo, Shintaro Seto, Minako Hijikata, and Naoto Keicho

Corresponding Author(s): Naoto Keicho, The Research Institute of Tuberculosis

Review Timeline:

Submission Date:	March 16, 2026
Editorial Decision:	April 19, 2026
Revision Received:	July 15, 2026
Editorial Decision:	July 31, 2026
Revision Received:	September 7, 2026
Accepted:	September 8, 2026

Editor: Sladjana Prisic

Reviewer(s): Disclosure of reviewer identity is with reference to reviewer comments included in decision letter(s). The following individuals involved in review of your submission have agreed to reveal their identity: Qinglan Wang (Reviewer #2)

Transaction Report:

(Note: With the exception of the correction of typographical or spelling errors that could be a source of ambiguity, letters and reports are not edited. The original formatting of letters and referee reports may not be reflected in this compilation.)

DOI: https://doi.org/10.1128/spectrum.00915-26

Re: Spectrum00915-26 (Deletion of *ppe27* in *Mycobacterium bovis* BCG augments macrophage immune responses during infection)

Dear Dr. Naoto Keicho:

Thank you for the privilege of reviewing your work. Below you will find my comments, instructions from the Spectrum editorial office, and the reviewer comments.

Please return the manuscript within 60 days; if you cannot complete the modification within this time period, please contact me. If you do not wish to modify the manuscript and prefer to submit it to another journal, notify me immediately so that the manuscript may be formally withdrawn from consideration by Spectrum.

Revision Guidelines
To submit your modified manuscript, log into the submission site at https://spectrum.msubmit.net/cgi-bin/main.plex. Go to Author Tasks and click the appropriate manuscript title to begin. The information you entered when you first submitted the paper will be displayed; update this as necessary. Note the following requirements:

• Upload point-by-point responses to the issues raised by the reviewers in a file named "Response to Reviewers," NOT in your cover letter.
• Upload a compare copy of the manuscript (without figures) as a "Marked-Up Manuscript" file.
• Upload a clean .DOC/.DOCX version of the revised manuscript and remove the previous version.
• Each figure must be uploaded as a separate, editable, high-resolution file (TIFF or EPS preferred), and any multipanel figures must be assembled into one file.
• Any supplemental material intended for posting by ASM should be uploaded with their legends separate from the main manuscript. You can combine all supplemental material into one file (preferred) or split it into a maximum of 10 files with all associated legends included.

For complete guidelines on revision requirements, see our Submission and Review Process webpage. Submission of a paper that does not conform to guidelines may delay acceptance of your manuscript.

Data availability: ASM policy requires that data be available to the public upon online posting of the article, so please verify all links to sequence records, if present, and make sure that each number retrieves the full record of the data. If a new accession number is not linked or a link is broken, provide Spectrum production staff with the correct URL for the record. If the accession numbers for new data are not publicly accessible before the expected online posting of the article, publication may be delayed; please contact production staff (Spectrum@asmusa.org) immediately with the expected release date.

Publication Fees: For information on publication fees and which article types are subject to charges, visit our website. If your manuscript is accepted for publication and any fees apply, you will be contacted separately about payment during the production process; please follow the instructions in that e-mail. Arrangements for payment must be made before your article is published.

ASM Membership: Corresponding authors may join or renew ASM membership to obtain discounts on publication fees. Need to upgrade your membership level? Please contact Customer Service at Service@asmusa.org.

The ASM Journals program strives for constant improvement in our submission and publication process. Please tell us how we can improve your experience by taking this quick Author Survey.

Thank you for submitting your paper to Spectrum.

Sincerely,
Sladjana Prisic
Editor
Microbiology Spectrum

Reviewer #1 (Comments for the Author):

Summary:

This study examines the impact of ppe27 deletion on tuberculosis pathogenesis during macrophage infection using M. bovis BCG as the model organism. To do so, the authors constructed a *ppe27* deletion mutant and did dual RNA-seq to examine both

the host and bacterial responses during infection. Their findings show that deletion of *ppe27* results in an increased inflammatory response and modulated enriched GSEA pathways in THP-1-derived macrophages infected with ppe27 deletion vs. wild-type BCG. Validation assays were also done using RT-qPCR and cytokine release assays to complement the RNA-seq data. Lastly, the effects of *ppe27* deletion on bacterial physiology were elucidated intracellular BCG bacteria, which showed that*ppe27* deletion resulted in few changes during macrophage infection. Overall, the manuscript provides further evidence that ppe27 may modulate the host response during infection.

The manuscript is within the scope of the mSpectrum journal because the authors' work was rigorous and their methodology was clear and sound. However, there are multiple facets that must be addressed to strengthen their findings and conclusions.

Major comments:

- Wild-type (wt) and *ppe27* deletion (Dppe27) strains of BCG were used for all experiments. However, a ppe27 complement strain was not generated nor used in this study, which is commonly done in the deletion mutant research. To confirm their observations with ppe27 are true, validation RT-qPCR experiments using the ppe27 complement strain must be done.

- DEG comparisons: host DEGs in wt-infected vs Dppe27-infected macrophages were determined, whereas the bacterial DEGs were done using Dppe27 vs wt BCG. For clarity and consistency, the comparisons should be Dppe27 vs wt BCG for both host and bacterial DEGs. Specifically, wt vs Dppe27 is shown in Figure 2D, whereas Dppe27 vs wt is shown in all other Figures.

-GSEA: enriched pathways were determined in wt-infected vs ppe27-infected and ppe27-infected vs wt-infected macrophages (Figure 3C and 3D). Given that the only difference between these two comparisons should be the direction of fold change (upregulated vs downregulated), it is presently unclear how GSEA was done for these comparisons.

-Cytokine assay: TNFa, IL-6 and IL-9 were claimed to be higher in Dppe27-infected vs. wt-infected and non-infected macrophages (lines 259-262). This is not in agreement with the panels in Figure 5 which show that the comparisons were statistically significant for only Dppe27-infected vs. non-infected macrophages.

-Bacterial RNA-seq and annotation: only one of the four DEGs, JTY_3006, was focused on in this analysis, while the other three were not discussed. Furthermore, JTY_3006 was mildly downregulated in Dppe27 vs wt BCG with a fold change of -1.7. Of the three undiscussed DEGs, JTY_3135 and sdhD3 were strongly upregulated in Dppe27 vs wt BCG with fold changes of 48 and 40, respectively. It would also be beneficial if the authors were to mention these genes in their discussion given the strong upregulation.

-Methods - bacterial culturing: the details regarding the medium and temperature used to grow BCG were included, but several other details are missing for culturing in liquid medium: the initial inoculum, the volume of medium (e.g., 50 mL), the total volume of the culture vessel (e.g., 250 mL), and if the cultures were shaken (and the speed, e.g., 100 rpm) or left at standing without shaking. These factors affect the oxygenation of the culture and, therefore, the physiology of the bacteria. The length of time for growth on solid 7H10 medium is not given as well, which includes the incubation time when growing and selecting for the *ppe27* deletion mutant. Including these details will improve the reproducibility of the data.

-Methods - infection preparation: the details regarding the preparation of BCG for macrophage infection are not currently described (e.g., harvesting, washing, removal of cluumps, resuspension medium/buffer). The method used to prepare the bacteria for infection is crucial because it has been shown that different preparation methods to remove clumps of bacteria can affect the mycobacterial cell wall and host response. See Mittal et al, 2023, doi: https://doi.org/10.7554/eLife.85416. Including these details will improve the reproducibility of the data.

-It would be beneficial to include CFU data of wt, Dppe27 and *ppe27* complement BCG from macrophages (and from the cell suspensions used to infect the macrophages at MOI 10). Although there has been previous work showing the prolonged survival of *ppe27* overexpression in M. smegmatis, survival data from this study's BCG Tokyo 172 strain would provide insight into the effects of ppe27 deletion on bacterial survival in this BCG strain and, potentially, complement its findings with heightened inflammatory response.

-Validation RT-qPCR of the bacterial RNA-seq data is needed to confirm the data (e.g., RT-qPCR of JTY_3006, *sdhD3*, etc).

Minor comments:

-Lines 251-252: the subheading is quite long and should be shortened.

-Lines 302-305: it is mistakenly stated that there were only 4 DEGs in broth-grown Dppe27 vs WT BCG and refers to Figure 7D. It should state for intracellular Dppe27 vs wt BCG and refer to Figure 7E.

-Line 305-307: it would be helpful to include JTY_3006 in this sentence because it is highlighted in Figure 7E.

-Line 525: a citation is needed for the ddCT-method.

-Figure 1: two of the three lines that should point at the cover slips point at the cells on the culture dish.

-Figure 2 and Figure 6: the axis label font is quite small on the DEG plots. Font size should be increased to improve readability.

-Figure 4 and Figure 5: the axis lines, significance bars, and standard deviation bars have different thicknesses. These should be modified so the thickness is consistent across all graphs (e.g., 1pt thickness).

-Figure 4: the non-infected conditions are missing error bars in each panel. They should be added because there should be variability in the non-infected macrophages.

-Methods: the p-value and fold change cutoffs are provided in the results section, but it would be beneficial to include the p-value and fold change cutoffs for DEGs in the methods section too.

Reviewer #2 (Comments for the Author):

This manuscript tackles an important question in mycobacterial host-pathogen interaction biology and has several strengths. In particular, the combined host-pathogen RNA-seq design is valuable, the mutant construction is carefully checked by long- and short-read genome sequencing, and the authors make a clear effort to validate transcriptomic observations with RT-qPCR and cytokine protein measurements. The finding that ppe27 deletion in BCG is associated with stronger TNF/IFN/inflammatory signatures is potentially interesting and could be relevant to attenuation and vaccine design. However, in its current form, the manuscript feels more preliminary than definitive, and several points should be addressed before the conclusions can be considered sufficiently supported for Microbiology Spectrum.

Major Comments

1. Lack of a complemented strain

A major limitation of the study is the absence of a genetic complementation strain. The conclusions are currently based on comparisons between wild-type BCG and the Δppe27 mutant alone. Without complementation, it is difficult to determine whether the observed host and bacterial phenotypes are specifically caused by loss of ppe27, rather than by unintended secondary effects associated with mutant construction or local perturbation of the ESX-5 locus. Inclusion of a complemented strain would be important to establish causality more convincingly.

2. Unclear exclusion of extracellular bacteria in the intracellular bacterial transcriptome analysis

It is not clear whether extracellular, non-internalized bacteria were sufficiently removed before isolation of bacterial RNA for the intracellular transcriptome analysis. For example, after infection of THP-1-derived macrophages, were extracellular bacteria removed by washing after 4-6 h, followed by antibiotic treatment to kill remaining extracellular bacilli? In addition, did the authors perform any quality control to quantify how many bacteria were truly intracellular versus extracellular (either free in the culture medium or attached to the macrophage surface)? This issue is particularly important for interpretation of the bacterial RNA-seq data, because contamination by extracellular bacteria could substantially affect the transcriptomic profile attributed to the intracellular population. This concern is even more relevant given that only a very limited number of genes were found to differ between intracellular wtBCG and Δppe27 bacteria.

3. Potential confounding effect of differences in bacterial uptake or intracellular burden

The study compares host and bacterial transcriptomes at 24 h post-infection between macrophages infected with wtBCG and those infected with Δppe27 BCG. However, it remains unclear whether the two strains are taken up by macrophages with equal efficiency, or whether they achieve comparable intracellular burdens at this time point. Differences in macrophage transcriptional responses could arise indirectly if Δppe27 affects phagocytic uptake, invasion efficiency, or intracellular survival, thereby altering bacterial load per cell. The authors should provide evidence that the transcriptomic differences observed in host cells are attributable to ppe27 deletion per se, rather than to differences in intracellular bacterial burden.

4. Possible indirect effects of ppe27 deletion on secretion of other ESX-5 substrates

Because ppe27 is located within the ESX-5 gene cluster, the possibility of broader effects on ESX-5-dependent secretion should be carefully considered. There is substantial evidence that secretion of ESX system substrates can be interdependent. Therefore, deletion of ppe27 may affect the secretion, surface localization, or stability of other ESX-5-associated substrates in BCG. If so, the host transcriptomic changes reported here may not be caused solely by loss of PPE27 itself, but could instead reflect altered export of other ESX-5 substrates, including additional PE/PPE proteins. This is an important mechanistic issue that should ideally be addressed experimentally. At minimum, it should be discussed explicitly as a key caveat in the interpretation of the data.

Minor Comments

1. Preparation of the mycobacterial inoculum should be described more clearly.
Because mycobacteria grown in vitro readily form clumps, it is important to clarify whether the bacterial inoculum used for macrophage infection consisted predominantly of single-cell suspensions. If so, the authors should describe in detail how this was achieved (for example, by repeated pipetting, through a syringe needle, low-speed centrifugation, or filtration). This information is important because bacterial clumping can substantially affect the effective multiplicity of infection, uptake efficiency, and reproducibility of host response measurements.

2. The validation of host transcriptomic findings could be expanded.
The current RT-qPCR validation focuses mainly on TNF-related and selected inflammatory genes, and the cytokine assay identifies increases in G-CSF, IL-10, TNF-α, IL-6, and IL-9. It would strengthen the manuscript if the authors provided a clearer rationale for the choice of validation targets and included one or two additional IFN-related markers, particularly because the manuscript emphasizes enrichment of both IFN-α and IFN-γ signaling pathways.

3. Statistical reporting should be made more transparent.
The manuscript states that three biological replicates were used and that one-way ANOVA followed by Tukey's multiple-comparison test was applied. However, for each assay, the authors should clearly define what constituted a biological replicate, whether the RNA-seq replicates were generated from infections performed on the same day or in fully independent experiments, and how batch effects were assessed and handled. Because the manuscript explicitly notes that batch variation limited DEG detection, readers need a more detailed explanation of how this issue was addressed analytically.

4. The manuscript would benefit from careful language editing.
There are multiple grammatical and phrasing issues throughout the text. A few examples include:

"its function during host-pathogen interactions remain poorly defined" should be "remains"
"To access the impact of ppe27 disruption" should be "assess"
"BCGΔppe27-infeced cells" should be "infected"
"animo acid" in Supplementary Figure 3 should be "amino acid"
"Normalized Enhancement Score" in the Figure 3 legend should be "Normalized Enrichment Score"
"subtract the background effect" is awkward and should be rewritten more precisely

A thorough revision by a fluent English speaker or professional editing service would improve readability.

5. Figure legends and the presentation of results could be improved.
The figure legends are generally understandable, but some panels would benefit from fuller explanation. For example, Figures 2 and 6 should state the comparison direction and significance thresholds more explicitly and in a more reader-friendly format. In addition, Figures 4 and 5 should clearly indicate the exact n values and specify whether the error bars represent SEM or SD, both in the figure legends and in the Methods section.

This manuscript tackles an important question in mycobacterial host–pathogen interaction biology and has several strengths. In particular, the combined host–pathogen RNA-seq design is valuable, the mutant construction is carefully checked by long- and short-read genome sequencing, and the authors make a clear effort to validate transcriptomic observations with RT-qPCR and cytokine protein measurements. The finding that ppe27 deletion in BCG is associated with stronger TNF/IFN/inflammatory signatures is potentially interesting and could be relevant to attenuation and vaccine design. However, in its current form, the manuscript feels more preliminary than definitive, and several points should be addressed before the conclusions can be considered sufficiently supported for Microbiology Spectrum.

Major Comments

1. Lack of a complemented strain

A major limitation of the study is the absence of a genetic complementation strain. The conclusions are currently based on comparisons between wild-type BCG and the Δppe27 mutant alone. Without complementation, it is difficult to determine whether the observed host and bacterial phenotypes are specifically caused by loss of ppe27, rather than by unintended secondary effects associated with mutant construction or local perturbation of the ESX-5 locus. Inclusion of a complemented strain would be important to establish causality more convincingly.

2. Unclear exclusion of extracellular bacteria in the intracellular bacterial transcriptome analysis

It is not clear whether extracellular, non-internalized bacteria were sufficiently removed before isolation of bacterial RNA for the intracellular transcriptome analysis. For example, after infection of THP-1-derived macrophages, were extracellular bacteria removed by washing after 4–6 h, followed by antibiotic treatment to kill remaining extracellular bacilli? In addition, did the authors perform any quality control to quantify how many bacteria were truly intracellular versus extracellular (either free in the culture medium or attached to the macrophage surface)? This issue is particularly important for interpretation of the bacterial RNA-seq data, because contamination by extracellular bacteria could substantially affect the transcriptomic profile attributed to the intracellular population. This concern is even more relevant given that only a very limited number of genes were found to differ between intracellular wtBCG and Δppe27 bacteria.

3. Potential confounding effect of differences in bacterial uptake or intracellular burden

The study compares host and bacterial transcriptomes at 24 h post-infection between macrophages infected with wtBCG and those infected with Δppe27 BCG. However, it remains unclear whether the two strains are taken up by macrophages with equal efficiency, or whether they achieve comparable intracellular burdens at this time point. Differences in macrophage transcriptional responses could arise indirectly if Δppe27 affects phagocytic uptake, invasion efficiency, or intracellular survival, thereby altering bacterial load per cell. The authors should provide evidence that the transcriptomic differences observed in host cells are attributable to ppe27 deletion per se, rather than to differences in intracellular bacterial burden.

4. Possible indirect effects of ppe27 deletion on secretion of other ESX-5 substrates

Because ppe27 is located within the ESX-5 gene cluster, the possibility of broader effects on ESX-5-dependent secretion should be carefully considered. There is substantial evidence that secretion of ESX system substrates can be interdependent. Therefore, deletion of ppe27 may affect the secretion, surface localization, or stability of other ESX-5-associated substrates in BCG. If so, the host transcriptomic changes reported here may not be caused solely by loss of PPE27 itself, but could instead reflect altered export of other ESX-5 substrates, including additional PE/PPE proteins. This is an important mechanistic issue that should ideally be addressed experimentally. At minimum, it should be discussed explicitly as a key caveat in the interpretation of the data.

Minor Comments

1. Preparation of the mycobacterial inoculum should be described more clearly.

Because mycobacteria grown in vitro readily form clumps, it is important to clarify whether the bacterial inoculum used for macrophage infection consisted predominantly of single-cell suspensions. If so, the authors should describe in detail how this was achieved (for example, by repeated pipetting, through a syringe needle, low-speed centrifugation, or filtration). This information is important because bacterial clumping can substantially affect the effective multiplicity of infection, uptake efficiency, and reproducibility of host response measurements.

2. The validation of host transcriptomic findings could be expanded.

The current RT-qPCR validation focuses mainly on TNF-related and selected inflammatory genes, and the cytokine assay identifies increases in G-CSF, IL-10, TNF-α, IL-6, and IL-9. It would strengthen the manuscript if the authors provided a clearer rationale for the choice of validation targets and included one or two additional IFN-related markers, particularly because the manuscript emphasizes enrichment of both IFN-α and IFN-γ signaling pathways.

3. Statistical reporting should be made more transparent.

The manuscript states that three biological replicates were used and that one-way ANOVA followed by Tukey's multiple-comparison test was applied. However, for each assay, the authors should clearly define what constituted a biological replicate, whether the RNA-seq replicates were generated from infections performed on the same day or in fully independent experiments, and how batch effects were assessed and handled. Because the manuscript explicitly notes that batch variation limited DEG detection, readers need a more detailed explanation of how this issue was addressed analytically.

4. The manuscript would benefit from careful language editing.

There are multiple grammatical and phrasing issues throughout the text. A few examples include:

"its function during host–pathogen interactions remain poorly defined" should be "remains"
"To access the impact of ppe27 disruption" should be "assess"
"BCGΔppe27-infeced cells" should be "infected"
"animo acid" in Supplementary Figure 3 should be "amino acid"
"Normalized Enhancement Score" in the Figure 3 legend should be "Normalized Enrichment Score"

“subtract the background effect” is awkward and should be rewritten more precisely

A thorough revision by a fluent English speaker or professional editing service would improve readability.

5. Figure legends and the presentation of results could be improved.

The figure legends are generally understandable, but some panels would benefit from fuller explanation. For example, Figures 2 and 6 should state the comparison direction and significance thresholds more explicitly and in a more reader-friendly format. In addition, Figures 4 and 5 should clearly indicate the exact n values and specify whether the error bars represent SEM or SD, both in the figure legends and in the Methods section.

Reviewer #1 (Comments for the Author):
Major comments:

- Wild-type (wt) and *ppe27* deletion (Dppe27) strains of BCG were used for all experiments. However, a ppe27 complement strain was not generated nor used in this study, which is commonly done in the deletion mutant research. To confirm their observations with ppe27 are true, validation RT-qPCR experiments using the ppe27 complement strain must be done.

Response:

We thank the reviewer for this insightful and constructive comment. We agree that genetic complementation is a well-established approach to confirm phenotypes observed in deletion mutants. We carefully considered performing a complementation analysis in this study. However, in the context of this study, we are concerned that complementation using either replicating or integrating vectors would not reliably restore the native wild-type phenotype for the following reasons:

1. A commonly used replicating vector, such as pMV261, is expected to drive substantially higher expression levels than those observed under native conditions. However, *ppe27* expression appears to require tight regulation to faithfully restore the wild-type phenotype. Overexpression of PPE27 has been reported to markedly enhance TNF-α production by macrophages while simultaneously promoting intracellular bacterial survival (DOI: 10.1089/jir.2016.0126). Given that TNF-α is a key host-protective cytokine that restricts bacteria growth (DOI 10.1016/j.immuni.2008.06.011), these findings suggest that PPE27 overexpression may induce complex and potentially confounding host-pathogen interactions that do not reflect the native biological function of the protein. Similarly, complementation of an *eccD5* mutant, which lacks a component of the ESX-5 secretion system, was unsuccessful when only the *eccD5* gene was introduced on a plasmid, highlighting the importance of native gene regulation within the ESX-5 locus (doi:10.1111/j.1365-2958.2012.08206.x).
2. Although integrating vectors such as pMV361 has been designed to allow stable chromosomal insertion, the integration site has been reported to be suboptimal for transcriptional activity, potentially resulting in insufficient gene expression (https://doi.org/10.1016/j.tube.2005.08.016). Consequently, successful complementation would depend heavily on promoter selection, integration site, and insertion orientation, each of which could substantially influence *ppe27* expression and complicate interpretation of the results. Furthermore, because *ppe27* is located

within the ESX-5 locus, its native genomic context is likely important for proper transcriptional regulation, and disruption of this context may prevent accurate restoration of the wild-type phenotype.

3. To address the possibility that the observed phenotype resulted from unintended mutations acquired during mutant construction, we performed whole-genome sequencing (WGS) of the Δ*ppe27* strain. WGS has become a robust and widely adopted approach for detecting secondary mutations in bacterial mutants and has been used in numerous recent studies to validate mutant strains (https://doi.org/10.1073/pnas.2509997122; https://doi.org/10.3390/antibiotics15010007 ; https://doi.org/10.1016/j.jbc.2025.110597 ; https://doi.org/10.3390/microorganisms12061120).
   Our WGS analysis did not identify additional mutations that could account for the observed phenotype, supporting the conclusion that the phenotypic differences are attributable to deletion of *ppe27*.

We nevertheless acknowledge that the absence of a complementation experiment is a limitation of this study. We have therefore added the following statement to the revised manuscript (lines 420-428):

> "We acknowledge the absence of a complementation study as a limitation of this work. As PPE27 expression appears to be tightly regulated, accurate restoration of its expression at levels and localization comparable to those of the wild-type strain would be essential for meaningful interpretation of any complementation phenotype. Following the approach described in a previous study (doi.org/10.1073/pnas.2509997122), we performed whole-genome sequencing to exclude the possibility of secondary mutations arising during the construction of the *ppe27* mutant. The absence of additional mutations that could account for the observed phenotype supports the conclusion that the phenotypic differences are attributable to the deletion of *ppe27*.

- DEG comparisons: host DEGs in wt-infected vs Dppe27-infected macrophages were determined, whereas the bacterial DEGs were done using Dppe27 vs wt BCG. For clarity and consistency, the comparisons should be Dppe27 vs wt BCG for both host and bacterial DEGs. Specifically, wt vs Dppe27 is shown in Figure 2D, whereas Dppe27 vs wt is shown in all other Figures.

Response:

We greatly thank the reviewer for pointing this out. We have modified Figure 2D to present the comparison as Δ*ppe27* vs. wt for consistency and have updated the corresponding text accordingly (lines 210-211).

-GSEA: enriched pathways were determined in wt-infected vs ppe27-infected and ppe27-infected vs wt-infected macrophages (Figure 3C and 3D). Given that the only difference between these two comparisons should be the direction of fold change (upregulated vs downregulated), it is presently unclear how GSEA was done for these comparisons.

Response:

We apologize for the lack of clarity in our description of the GSEA analysis.
GSEA was performed using the software developed by the Broad Institute as described in the Materials and Methods. The analysis indicated that 28/50 gene sets were upregulated/enriched in Δ*ppe27*-infected macrophages (7 gene sets are significant at FDR q-value; Supplementary Table 4C), while 22/50 gene sets were upregulated/enriched in wt-infected macrophages (only 1 gene set is significant at FDR q-value; Supplementary Table 4D).
It does suggest that Oxidative Phosphorylation in Δ*ppe27*-infected vs wt-infected macrophages was downregulated (NES=-1.95). Conversely, Oxidative Phosphorylation pathway in wt-infected vs Δ*ppe27*-infected macrophages was upregulated.

To improve data readability, we revised the figure and presented the results as horizontal bar plots (Figure 3A to 3C).

-Cytokine assay: TNFa, IL-6 and IL-9 were claimed to be higher in Dppe27-infected vs. wt-infected and non-infected macrophages (lines 259-262). This is not in agreement with the panels in Figure 5 which show that the comparisons were statistically significant for only Dppe27-infected vs. non-infected macrophages.

Response:

We apologize for the inconsistency between the statistical results and their description. In our initial analysis, we performed pairwise comparisons between groups using an unpaired t-test, which indicated a significant difference between Δ*ppe27*-infected and wt-infected macrophages. When a more conservative approach (one-way ANOVA) was applied to account for multiple comparisons, these significances were not observed. We have modified the description accordingly and revised text in lines 260–265.

-Bacterial RNA-seq and annotation: only one of the four DEGs, JTY_3006, was focused on in this analysis, while the other three were not discussed. Furthermore, JTY_3006 was mildly downregulated in Dppe27 vs wt BCG with a fold change of -1.7. Of the three undiscussed DEGs, JTY_3135 and sdhD3 were strongly upregulated in Dppe27 vs wt BCG with fold changes of 48 and 40, respectively. It would also be beneficial if the authors were to mention these genes in their discussion given the strong upregulation.

Response:

We thank the reviewer for this comment.

Indeed, we initially identified four DEGs, of which *JTY_3135* and *sdhD3* were strongly upregulated in Δ*ppe27* vs wt. However, closer inspection revealed that sequencing reads corresponding to these genes mapped to multiple genomic locations. *JTY_3135* is transposase IS*1081* sequence, and BCG Tokyo 172 has five copies on its genome. BCG Tokyo 172 has triple tandem duplications of the genomic region including the genes from *astB* to *sdhD*, and there are three identical *sdhD3* sequences (doi: 10.1186/s12864-016-3355-1). We have confirmed that the numbers and locations of IS*1081* and *sdhD* genes were identical and not changed between wtBCG and the Δ*ppe27* strains by whole genome sequencing using both short and long read sequencing. When multimapping of reads was allowed (ten times at the most), reads were mapped randomly to the repetitive sequences, but expression levels of the other identical IS*1081* (JTY_1077, JTY_1234, JTY_2526, JTY_3041) and *sdhD* genes (*sdhD_1*and *sdhD_2*) were not significantly different. Moreover, when the mapping was restricted to only once, the expression levels of *JTY_3135* and *sdhD3* were not significantly different. Given the above abundance of repetitive sequences in the BCG Tokyo 172 genome, such multi-mapping is likely to introduce mapping-related quantification bias, particularly in short-read sequencing approaches.

For *JTY_0110* gene, the reads were uniquely aligned to the BCG genome. However, the RNA level of *JTY_0110* was just above the detection threshold by RT-qPCR (the new result is included in the Supplementary Figure 4). We have accordingly added these descriptions in the Result section (lines 306-319).

-Methods - bacterial culturing: the details regarding the medium and temperature used to grow BCG were included, but several other details are missing for culturing in liquid medium: the initial inoculum, the volume of medium (e.g., 50 mL), the total volume of the culture vessel (e.g., 250 mL), and if the cultures were shaken (and the speed, e.g., 100 rpm) or left at standing without shaking. These

factors affect the oxygenation of the culture and, therefore, the physiology of the bacteria. The length of time for growth on solid 7H10 medium is not given as well, which includes the incubation time when growing and selecting for the *ppe27* deletion mutant. Including these details will improve the reproducibility of the data.

Response:

We thank the reviewer for this suggestion and have added detailed information on bacterial culture conditions to the Materials and Methods section (lines 438-443).

-Methods - infection preparation: the details regarding the preparation of BCG for macrophage infection are not currently described (e.g., harvesting, washing, removal of clumps, resuspension medium/buffer). The method used to prepare the bacteria for infection is crucial because it has been shown that different preparation methods to remove clumps of bacteria can affect the mycobacterial cell wall and host response. See Mittal et al, 2023, doi: https://doi.org/10.7554/eLife.85416. Including these details will improve the reproducibility of the data.

Response:

We appreciate the reviewer for this valuable suggestion. We have added the detailed information in Materials and Methods section (lines 443-446).

-It would be beneficial to include CFU data of wt, Dppe27 and *ppe27* complement BCG from macrophages (and from the cell suspensions used to infect the macrophages at MOI 10). Although there has been previous work showing the prolonged survival of *ppe27* overexpression in M. smegmatis, survival data from this study's BCG Tokyo 172 strain would provide insight into the effects of ppe27 deletion on bacterial survival in this BCG strain and, potentially, complement its findings with heightened inflammatory response.

Response:

We thank the reviewer for this helpful comment. The CFU values of the bacterial suspensions used for macrophage infection have now been included in the Methods section (lines 483-484). In addition, we performed an independent infection experiment to quantify the macrophage-associated bacterial burden at 24 hpi. The results showed no significant difference in CFU counts between the Δ*ppe27* strain and wtBCG at 24 hpi (Supplementary Figure 5), although the Δ*ppe27* strain appeared to be internalized more efficiently by macrophages at 4 hpi (Supplementary Figure 6).

We have incorporated these results into the revised manuscript (lines 325-329).

-Validation RT-qPCR of the bacterial RNA-seq data is needed to confirm the data (e.g., RT-qPCR of JTY_3006, *sdhD3*, etc).

Response:

We agree with the reviewer's suggestion and performed an additional RT-qPCR analysis to validate the downregulation of *JTY_3006* in the Δ*ppe27* mutant. The results have been added to Figure 7E and the Results section (lines 307-319). As described above, *JTY_3135* and *sdhD3,* which were initially identified as differentially expressed by RNA-seq, were subsequently determined to be false positives because the sequencing reads mapped to multiple genomic locations. For *JTY_0110*, the transcript level detected by RT-qPCR was only marginally above the detection threshold. We therefore consider the data insufficient to support a reliable interpretation of its differential expression.

Minor comments:

-Lines 251-252: the subheading is quite long and should be shortened.

Response:

Following the reviewer's advice, the subheading has been shortened (line 255):

"Enhanced cytokine production in BCGΔ*ppe27*-infected cells"

-Lines 302-305: it is mistakenly stated that there were only 4 DEGs in broth-grown Dppe27 vs WT BCG and refers to Figure 7D. It should state for intracellular Dppe27 vs wt BCG and refer to Figure 7E.

Response:

We thank the reviewer for this careful observation. We have corrected the text from "broth-grown" to "macrophage-associated". Figure 6E and 7D are the correct corresponding figures for this statement and we have revised accordingly (line 306-309).

-Line 305-307: it would be helpful to include JTY_3006 in this sentence because it is highlighted in Figure 7E.

Response:

We appreciate the reviewer for this suggestion. As we have revised the paragraph, we now mentioned *JTY_3006* in line 319.

-Line 525: a citation is needed for the ddCT-method.

Response:

We thank the reviewer for this suggestion. A citation for the ddCT-method has been added to the Methods section (lines 558-560).

-Figure 1: two of the three lines that should point at the cover slips point at the cells on the culture dish.

Response:

We appreciate the reviewer's careful evaluation. The figure has been revised.

-Figure 2 and Figure 6: the axis label font is quite small on the DEG plots. Font size should be increased to improve readability.

Response:

We agree with the reviewer's suggestion and have enlarged the axis label font on the DEG plots.

-Figure 4 and Figure 5: the axis lines, significance bars, and standard deviation bars have different thicknesses. These should be modified so the thickness is consistent across all graphs (e.g., 1pt thickness).

Response:

We appreciate this comment and have incorporated the suggested changes.

-Figure 4: the non-infected conditions are missing error bars in each panel. They should be added because there should be variability in the non-infected macrophages.

Response:

We thank the reviewer for this helpful comment. Error bars for the non-infected cells have been added to each panel of Figure 4.

-**Methods: the p-value and fold change cutoffs are provided in the results section, but it would be beneficial to include the p-value and fold change cutoffs for DEGs in the methods section too.**

Response:

We thank the reviewer for this suggestion. These details have been added to the Materials and Methods section (line 536).

Reviewer #2 (Comments for the Author):

Major Comments

1. Lack of a complemented strain

A major limitation of the study is the absence of a genetic complementation strain. The conclusions are currently based on comparisons between wild-type BCG and the Δppe27 mutant alone. Without complementation, it is difficult to determine whether the observed host and bacterial phenotypes are specifically caused by loss of ppe27, rather than by unintended secondary effects associated with mutant construction or local perturbation of the ESX-5 locus. Inclusion of a complemented strain would be important to establish causality more convincingly.

Response:

We thank the reviewer for this insightful remark. We agree that genetic complementation is a well-established approach to confirm phenotypes observed in deletion mutants. We carefully considered performing a complementation analysis in this study. However, in the context of this study, we are concerned that complementation using either replicating or integrating vectors would not reliably restore the native wild-type phenotype for the following reasons:

1. A commonly used replicating vector, such as pMV261, is expected to drive substantially higher expression levels than those observed under native conditions. However, *ppe27* expression appears to require tight regulation to faithfully restore the wild-type phenotype. Overexpression of PPE27 has been reported to markedly enhance TNF-α production by macrophages while simultaneously promoting intracellular bacterial survival (DOI: 10.1089/jir.2016.0126). Given that TNF-α is a key host-protective cytokine that restricts bacteria growth (DOI 10.1016/j.immuni.2008.06.011), these findings suggest that PPE27 overexpression may induce complex and potentially confounding host-pathogen interactions that do not reflect the native biological function of the protein. Similarly, complementation of an *eccD5* mutant, which lacks a component of the ESX-5 secretion system, was unsuccessful when only the *eccD5* gene was introduced on a plasmid, highlighting the importance of native gene regulation within the ESX-5 locus (doi:10.1111/j.1365-2958.2012.08206.x).
2. Although integrating vectors such as pMV361 has been designed to allow stable chromosomal insertion, the integration site has been reported to be suboptimal for transcriptional activity, potentially resulting in insufficient gene expression (https://doi.org/10.1016/j.tube.2005.08.016). Consequently, successful complementation would depend heavily on promoter selection, integration site, and insertion orientation, each of which could substantially influence *ppe27* expression

and complicate interpretation of the results. Furthermore, because *ppe27* is located within the ESX-5 locus, its native genomic context is likely important for proper transcriptional regulation, and disruption of this context may prevent accurate restoration of the wild-type phenotype.

3. In parallel, we sought to address the possibility of unintended secondary mutations in the Δ*ppe27* strain. Whole-genome sequencing (WGS) has become a robust and widely adopted approach for detecting off-target mutations introduced during mutant construction, and has been successfully applied in multiple studies to validate mutant strains (https://doi.org/10.3390/antibiotics15010007 ; https://doi.org/10.1016/j.jbc.2025.110597 ; https://doi.org/10.3390/microorganisms12061120). Accordingly, we performed WGS analysis to exclude the possibility that the observed phenotype of Δ*ppe27* resulted from unintended mutations elsewhere in the genome.
4. We reasoned that if deletion of *ppe27* perturbed the ESX-5 locus at the transcriptional level, such effects would likely be reflected in the RNA-seq dataset. However, our analysis did not identify any differential expressed genes associated with ESX-5 locus. Therefore, our data do not support the notion that *ppe27* deletion alters the transcription of other ESX-5-associated genes. We cannot exclude the possibility that deletion of *ppe27* may influence the secretion of function of other ESX-5 substrates at the post-transcriptional level. However, addressing this question would require extensive additional studies and is beyond the scope of the present work.

We nevertheless acknowledge that the absence of a complementation experiment is a limitation of this study. We have therefore added the following statement to the revised manuscript (lines 420-428):

> "We acknowledge the absence of a complementation study as a limitation of this work. As PPE27 expression appears to be tightly regulated, accurate restoration of its expression at levels and localization comparable to those of the wild-type strain would be essential for meaningful interpretation of any complementation phenotype. Following the approach described in a previous study (doi.org/10.1073/pnas.2509997122), we performed whole-genome sequencing to exclude the possibility of secondary mutations arising during the construction of the *ppe27* mutant. The absence of additional mutations that could account for the

observed phenotype supports the conclusion that the phenotypic differences are attributable to the deletion of *ppe27*."

.

2. Unclear exclusion of extracellular bacteria in the intracellular bacterial transcriptome analysis

It is not clear whether extracellular, non-internalized bacteria were sufficiently removed before isolation of bacterial RNA for the intracellular transcriptome analysis. For example, after infection of THP-1-derived macrophages, were extracellular bacteria removed by washing after 4-6 h, followed by antibiotic treatment to kill remaining extracellular bacilli? In addition, did the authors perform any quality control to quantify how many bacteria were truly intracellular versus extracellular (either free in the culture medium or attached to the macrophage surface)? This issue is particularly important for interpretation of the bacterial RNA-seq data, because contamination by extracellular bacteria could substantially affect the transcriptomic profile attributed to the intracellular population. This concern is even more relevant given that only a very limited number of genes were found to differ between intracellular wtBCG and Δppe27 bacteria.

Response:

We appreciate this comment and understand the concern.

In our study, macrophages were washed to remove extracellular bacteria before harvesting, without treating with antibiotics. This experimental design was based on previous reports showing that pinocytosed aminoglycosides can access macrophage phagosomes and exert intracellular antimicrobial activity (https://doi.org/10.1172/JCI57235). Consistent with this, some researchers have recommended avoiding post-infection washing and antibiotic treatment to minimize potential confounding effects (https://doi.org/10.3389/ fimmu.2026.1695558).

To directly address this concern, we performed additional CFU assays to evaluate the effects of post-infection washing and gentamicin treatment on intracellular bacterial burden. The experiments were conducted under the following three conditions (Figures shown below):

(1) **No early washing**: Infected macrophages were incubated for 24 hpi without washing. Cells were then washed three times immediately before harvesting intracellular bacteria for CFU enumeration at 24 hpi.

(2) **Early washing plus gentamicin treatment**: Infected macrophages were washed at

4 hpi and treated with gentamicin (100µg/mL) for 2 hours, followed by an additional washing step. Intracellular bacteria were harvested at 24 hpi for CFU enumeration.

(3) **Early harvesting at 4hpi**: Infected macrophages were washed at 4 hpi, followed by harvesting intracellular bacteria for CFU enumeration.

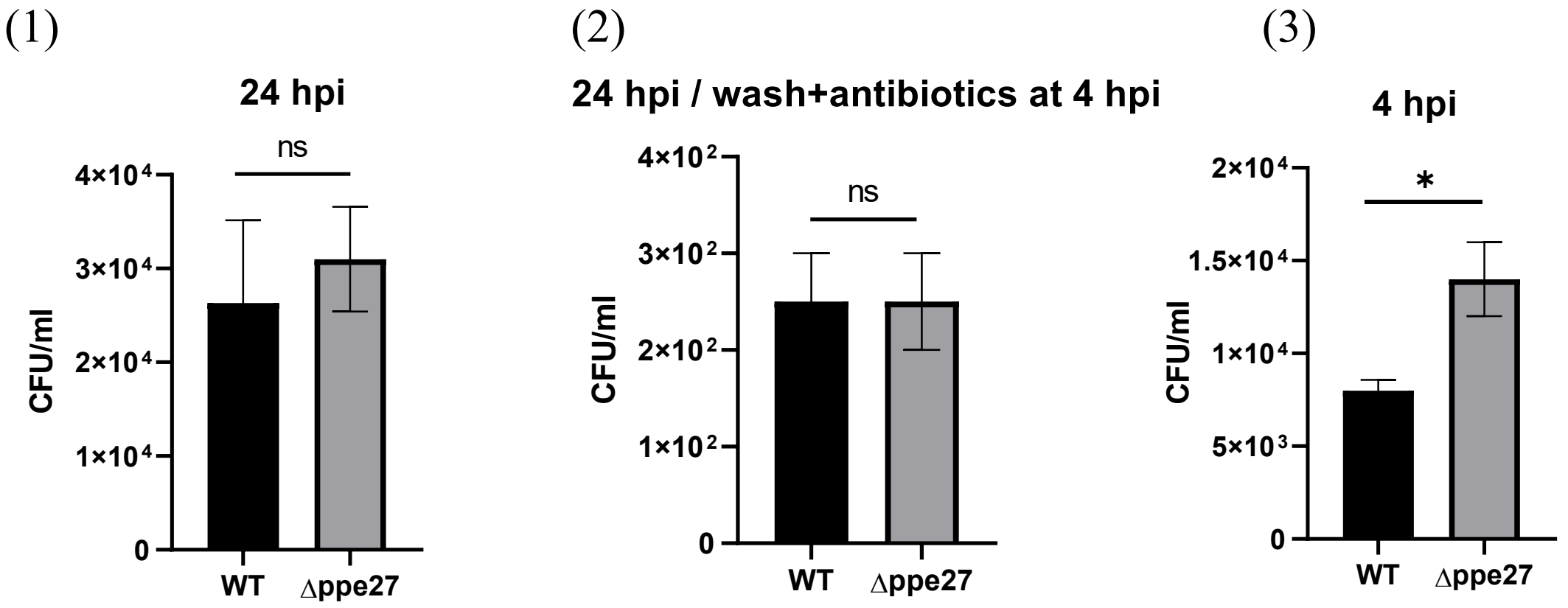

As shown in the figures above, early washing followed by gentamicin treatment at 4 hpi (Condition 2) resulted in approximately a 100-fold lower number of cell-associated bacteria at 24 hpi than the no-early-washing condition (Condition 1). This indicates that, under our experimental conditions, early washing and gentamicin treatment substantially reduced the number of bacteria recovered from macrophages. Therefore, Condition 2 cannot be used to demonstrate that the recovered bacteria were exclusively intracellular. In both Conditions 1 and 3, washing removed most extracellular planktonic bacteria from the culture medium.

We also acknowledge that, under the current experimental conditions, we cannot completely exclude the possibility that tightly surface-adhered bacteria were co-harvested. Therefore, we consider that the term "macrophage-associated bacterial RNA" is more appropriate than "intracellular bacterial RNA."

Nevertheless, we believe that following repeated washing, most non-adherent extracellular bacteria were removed. The remaining bacteria were predominantly cell-associated, consisting primarily of intracellular bacteria, although a small number of surface-attached bacteria may have remained. This interpretation is supported by the distinct transcriptional profiles observed between macrophage-associated bacteria and broth-grown bacteria, as shown in Figures 6B and 6C. If the macrophage-associated bacterial RNA samples had been substantially contaminated with extracellular bacterial RNA, the transcriptional profiles would be expected to more closely resemble those of

broth-grown bacteria. Instead, the marked transcriptional differences observed indicate that contamination by extracellular bacterial RNA, if present, was minimal. Thus, these findings provide additional indirect evidence that the washing procedure immediately before cell harvest was sufficient to remove the majority of extracellular bacteria.

Accordingly, we have revised the terminology throughout the manuscript, replacing "intracellular" with "macrophage-associated" where appropriate. In addition, we have included Figures of condition 1 and condition 3 as Supplementary Figure 5 and Supplementary Figure 6, respectively.

Potential confounding effect of differences in bacterial uptake or intracellular burden

The study compares host and bacterial transcriptomes at 24 h post-infection between macrophages infected with wtBCG and those infected with Δppe27 BCG. However, it remains unclear whether the two strains are taken up by macrophages with equal efficiency, or whether they achieve comparable intracellular burdens at this time point. Differences in macrophage transcriptional responses could arise indirectly if Δppe27 affects phagocytic uptake, invasion efficiency, or intracellular survival, thereby altering bacterial load per cell. The authors should provide evidence that the transcriptomic differences observed in host cells are attributable to ppe27 deletion per se, rather than to differences in intracellular bacterial burden.

Response:

We thank the reviewer for raising this important point. As described above, we performed an additional experiment to quantify the macrophage-associated bacterial burden at an early time point (4 hpi) to compare the uptake efficiency of the two strains by macrophages. The result showed that the Δ*ppe27* strain exhibited a higher bacterial burden than wtBCG at 4 hpi, indicating the Δ*ppe27* strain is internalized more efficiently by macrophages (Figure above: condition 3). However, by 24 hpi, the bacterial burdens of the two strains were comparable (Figure above: condition 1). As our host transcriptomic analysis was performed at 24 hpi rather than at the early uptake stage, we believe that the observed differences in host gene expression primarily reflect the macrophage responses at 24 hpi, rather than differences in the initial bacterial uptake.

4. Possible indirect effects of ppe27 deletion on secretion of other ESX-5 substrates

Because ppe27 is located within the ESX-5 gene cluster, the possibility of broader effects on ESX-5-dependent secretion should be carefully considered. There is substantial evidence that secretion of ESX system substrates can be interdependent. Therefore, deletion of ppe27 may affect the secretion, surface localization, or stability of other ESX-5-associated substrates in BCG. If so, the host transcriptomic changes reported here may not be caused solely by loss of PPE27 itself, but could instead reflect altered export of other ESX-5 substrates, including additional PE/PPE proteins. This is an important mechanistic issue that should ideally be addressed experimentally. At minimum, it should be discussed explicitly as a key caveat in the interpretation of the data.

Response:

We thank the reviewer for this insightful comment. Indeed, we cannot exclude the possibility that deletion of *ppe27* may indirectly affect the secretion of other ESX-5 associated substrates. While experimental validation of ESX-5-dependent secretion was beyond the scope of the present study, we have acknowledged this point as an important caveat in the Discussion section (lines 383–385).

Minor Comments

1. Preparation of the mycobacterial inoculum should be described more clearly. Because mycobacteria grown in vitro readily form clumps, it is important to clarify whether the bacterial inoculum used for macrophage infection consisted predominantly of single-cell suspensions. If so, the authors should describe in detail how this was achieved (for example, by repeated pipetting, through a syringe needle, low-speed centrifugation, or filtration). This information is important because bacterial clumping can substantially affect the effective multiplicity of infection, uptake efficiency, and reproducibility of host response measurements.

Response:

We thank the reviewer for bringing this to our attention. We have added the detailed information regarding bacterial inoculum in the Methods section (lines 443-445) and also cited our previously published paper.

2. The validation of host transcriptomic findings could be expanded.
The current RT-qPCR validation focuses mainly on TNF-related and selected inflammatory genes, and the cytokine assay identifies increases in G-CSF, IL-10, TNF-α, IL-6, and IL-9. It would strengthen the manuscript if the authors provided a clearer rationale for the choice of validation targets and included one or two

additional IFN-related markers, particularly because the manuscript emphasizes enrichment of both IFN-α and IFN-γ signaling pathways.

Response:

We are grateful to the reviewer for these insightful comments and suggestions. We reanalyzed the RNA-seq data using pairwise comparison and identified 45 DEGs between the wtBCG- and Δ*ppe27*-infected macrophages (Supplementary Table 3), including several genes involved in the IFN-α and IFN-γ signaling pathways. Based on the results, we then performed additional RT-qPCR analyses, which confirmed the upregulation of four additional IFN related genes, *IFI44*, *IFI44L*, *STAT4*, and *GBP1* in Δ*ppe27*-infected macrophages. These new validations have been incorporated into Figure 4 and are described in the Results section (lines 249-253), Methods section (lines 551-560) and Discussion section (lines 365-375).

Statistical reporting should be made more transparent.

The manuscript states that three biological replicates were used and that one-way ANOVA followed by Tukey's multiple-comparison test was applied. However, for each assay, the authors should clearly define what constituted a biological replicate, whether the RNA-seq replicates were generated from infections performed on the same day or in fully independent experiments, and how batch effects were assessed and handled. Because the manuscript explicitly notes that batch variation limited DEG detection, readers need a more detailed explanation of how this issue was addressed analytically.

Response:

We thank the reviewer for this insightful suggestion. We have added the following description to the Methods section (Lines 487-491).

"A biological replicate was defined as an independent experiment encompassing infection, RNA isolation, library preparation, and RNA sequencing. This workflow—from infection through sequencing—was repeated to generate second and then third replicates performed on different days. Thus, all three replicates were derived from fully independent experiments."

In addition, batch effects were accounted for by including a "batch" parameter during RNA-seq analysis in CLC Genomics Workbench (lines 535-536).

4. The manuscript would benefit from careful language editing.

There are multiple grammatical and phrasing issues throughout the text. A few examples include:

"its function during host-pathogen interactions remain poorly defined" should be "remains"
"To access the impact of ppe27 disruption" should be "assess"
"BCGΔppe27-infeced cells" should be "infected"
"animo acid" in Supplementary Figure 3 should be "amino acid"
"Normalized Enhancement Score" in the Figure 3 legend should be "Normalized Enrichment Score"

Response:

We deeply apologize for the overlooked mistakes. We have corrected the typographical errors accordingly.

"subtract the background effect" is awkward and should be rewritten more precisely

Response:

We thank the reviewer for pointing this out. The sentence has been revised (line 463).

A thorough revision by a fluent English speaker or professional editing service would improve readability.

Response:

We thank the reviewer for this helpful suggestion. The manuscript has been carefully proofread by a professional editing service, and typographical errors have been corrected throughout the manuscript.

5. Figure legends and the presentation of results could be improved.
The figure legends are generally understandable, but some panels would benefit from fuller explanation. For example, Figures 2 and 6 should state the comparison direction and significance thresholds more explicitly and in a more reader-friendly format. In addition, Figures 4 and 5 should clearly indicate the exact n values and specify whether the error bars represent SEM or SD, both in the figure legends and in the Methods section.

Response:

We thank the reviewer for this helpful suggestion. We have revised the figure legends for better explanations.

Re: Spectrum00915-26R1 (Deletion of *ppe27* in *Mycobacterium bovis* BCG augments macrophage immune responses during infection)

Dear Dr. Naoto Keicho:

Thank you for the privilege of reviewing your work. Below you will find my comments, instructions from the Spectrum editorial office, and the reviewer comments.

Since both reviewers thought that the lack of complementation is a serious problem that was not fixed with this resubmission, I initially decided to reject your paper. However, I changed my mind and I will allow another modification, but you must tone down your conclusions and emphasize the lack of complementation as a weakness of your study. Please address the rest of the concerns from the reviewers.

Please return the manuscript within 60 days; if you cannot complete the modification within this time period, please contact me. If you do not wish to modify the manuscript and prefer to submit it to another journal, notify me immediately so that the manuscript may be formally withdrawn from consideration by Spectrum.

Revision Guidelines
To submit your modified manuscript, log into the submission site at https://spectrum.msubmit.net/cgi-bin/main.plex. Go to Author Tasks and click the appropriate manuscript title to begin. The information you entered when you first submitted the paper will be displayed; update this as necessary. Note the following requirements:

• Upload point-by-point responses to the issues raised by the reviewers in a file named "Response to Reviewers," NOT in your cover letter.
• Upload a compare copy of the manuscript (without figures) as a "Marked-Up Manuscript" file.
• Upload a clean .DOC/.DOCX version of the revised manuscript and remove the previous version.
• Each figure must be uploaded as a separate, editable, high-resolution file (TIFF or EPS preferred), and any multipanel figures must be assembled into one file.
• Any supplemental material intended for posting by ASM should be uploaded with their legends separate from the main manuscript. You can combine all supplemental material into one file (preferred) or split it into a maximum of 10 files with all associated legends included.

For complete guidelines on revision requirements, see our Submission and Review Process webpage. Submission of a paper that does not conform to guidelines may delay acceptance of your manuscript.

Data availability: ASM policy requires that data be available to the public upon online posting of the article, so please verify all links to sequence records, if present, and make sure that each number retrieves the full record of the data. If a new accession number is not linked or a link is broken, provide Spectrum production staff with the correct URL for the record. If the accession numbers for new data are not publicly accessible before the expected online posting of the article, publication may be delayed; please contact production staff (Spectrum@asmusa.org) immediately with the expected release date.

Publication Fees: For information on publication fees and which article types are subject to charges, visit our website. If your manuscript is accepted for publication and any fees apply, you will be contacted separately about payment during the production process; please follow the instructions in that e-mail. Arrangements for payment must be made before your article is published.

ASM Membership: Corresponding authors may join or renew ASM membership to obtain discounts on publication fees. Need to upgrade your membership level? Please contact Customer Service at Service@asmusa.org.

The ASM Journals program strives for constant improvement in our submission and publication process. Please tell us how we can improve your experience by taking this quick Author Survey.

Thank you for submitting your paper to Spectrum.

Sincerely,
Sladjana Prisic
Editor
Microbiology Spectrum

Reviewer #1 (Comments for the Author):

Thank you to the authors for addressing all of our comments and suggestions.

Comments:
- The authors responded to the lack of a complement strain by highlighting their WGS data and modifying their concluding paragraph.
- The authors have appropriately addressed my comments about the direction of their comparisons and the associated figures (e.g., ppe27 mutant vs wt).
- The authors have appropriately addressed my comment about the GSEA section.
- The authors have appropriately addressed the cytokine significance issue.
- The authors have appropriately clarified the four DEGs and the issue with gene duplication regarding JTY_3135 in their response and in their manuscript (e.g., line 311-314).
- The authors have improved the reproducibility of their incubation conditions and infection assay by providing the necessary details.
- The authors have included the requested CFU data and RT-PCR results along with NEW infection CFUs.
- The authors addressed the minor comments.
- The CFU data showing increased uptake and/or association of ppe27 vs wt bacteria at 4 hpi is very interesting. The authors' data with gentamicin also highlights the disadvantage of using antibiotics to "select" for intracellular bacteria.

Suggestions:
- Inclusion of the WGS data as a supplementary table would strengthen their conclusion because it is not included in the manuscript, other than Supplementary Figure 2 which shows the disrupted gene locus. For example, a table with the SNPs and other mutations from the wt and ppe27 mutant strains could be included (e.g., ppe27 compared to wt; both compared to the reference genome).
- The lack of a complement strain remains a pressing issue. The authors are correct that complementation can be problematic and difficult in Mycobacterium tuberculosis (MTB); however, there are numerous studies that have complemented MTB genes, regardless of the effects of the surrounding DNA sequence (i.e., genomic context). While the overexpression of ppe27 in Mycobacterium smegmatis led to increased production of TNFa, the same may not be observed in MTB. Furthermore, there are methods of controlling and/or titrating the expression of genes in MTB that may help to prevent overexpression, if it an issue (e.g., Tet- or nitrile-inducible promoter). Addressing this issue would greatly improve the study's validity.

Reviewer #2 (Comments for the Author):

I appreciate the authors' efforts to revise the manuscript. The revised version is improved by the addition of culture and infection-preparation details, clarification of DEG comparison directions, additional RT-qPCR validation including IFN-related genes, JTY_3006 validation, CFU data, and more transparent statistical descriptions. The central observation that BCGΔppe27 infection is associated with enhanced inflammatory and IFN-related responses in THP-1-derived macrophages is generally supported by the presented RNA-seq, RT-qPCR, and cytokine data.
However, several important issues remain and should be addressed before publication.

1. The lack of a complemented strain remains a major limitation. The authors' explanation that complementation may be difficult because PPE27 expression requires native regulation is understandable, but not fully convincing. In the PE/PPE field, multiple previous studies, including work on PPE51/PE19, PPE51-mediated nutrient uptake, PE15/PPE20, and other PE/PPE proteins, have generated complemented strains or used functional complementation approaches to support causality (PMID: 32139546,32138343, 36441815, 42031738). Therefore, the general argument that PE/PPE complementation is unreliable or infeasible is too broad. If the authors cannot provide a complemented strain, they should explicitly acknowledge that causal attribution to ppe27 remains incomplete.

2. WGS is useful but cannot substitute for complementation. The revised manuscript states that long- and short-read WGS confirmed the intended 476-bp deletion and did not identify off-target mutations. However, the manuscript does not provide sufficient detail on sequencing depth, minor-variant thresholds, structural-variant calling, copy-number analysis, or how repetitive PE/PPE regions were resolved. This is particularly important because PE/PPE loci are repetitive and difficult to analyze, and low-frequency variants or structural changes may not be confidently excluded. The authors should soften statements implying that WGS fully rules out other genetic explanations.

3. The conclusions remain too strong. Statements such as "PPE27 is a negative regulator of host inflammatory responses," "PPE27 is not just an antigen but an active immune-modulating virulence factor," and "loss of a single PPE molecule causes a clear phenotype" should be revised. More appropriate wording would be: "ppe27 deletion is associated with enhanced macrophage inflammatory responses" or "PPE27 may contribute to modulation of host inflammatory responses."
4. The term "macrophage-associated bacterial RNA" should be used consistently. The authors appropriately changed much of the terminology from "intracellular" to "macrophage-associated," but some wording still implies a strictly intracellular bacterial population, such as "within host cells," "remove non-internalized bacilli," "bacteria residing in the host cells," and "internalized more efficiently." Because tightly surface-adhered bacteria cannot be excluded, these phrases should be revised.
5. The new CFU data are useful, especially the finding that bacterial burdens are comparable at 24 hpi. However, the higher Δppe27 burden at 4 hpi indicates that the two strains are not taken up or retained identically at early time points. Even if burdens

are similar at 24 hpi, the host transcriptome at 24 hpi may still reflect different early exposure histories, different bacteria-per-cell distributions, or altered attachment/internalization dynamics. The authors should explicitly state this limitation. The conclusion should be framed as macrophage responses associated with Δppe27 infection under the tested conditions, rather than as a direct effect of PPE27 loss independent of bacterial uptake dynamics.

6. Only four DEGs were detected between wtBCG and BCGΔppe27 under macrophage-associated conditions, and two of the initially prominent candidates appear to be affected by multi-mapping. The validation of JTY_3006 is useful, but the link between PPE27, JTY_3006, SAM-dependent methyltransferase activity, and host epigenetic regulation remains speculative. The manuscript should clearly state that JTY_3006 is a candidate transcriptional change and that no direct evidence currently connects JTY_3006 to host epigenetic modification or the observed inflammatory phenotype.

7. The revised Methods state that batch effects were accounted for by including a batch parameter in CLC Genomics Workbench. Please provide more detail on the statistical model/design formula, normalization method, whether the infection-day batch was paired across groups, and how the small sample size was handled. For GSEA, please specify the exact ranking metric, FDR threshold used to define significance, and whether all plotted pathways are significant or selected for visualization.

Reviewer #1 (Comments for the Author):

Thank you to the authors for addressing all of our comments and suggestions.

Comments:

- The authors responded to the lack of a complement strain by highlighting their WGS data and modifying their concluding paragraph.
- The authors have appropriately addressed my comments about the direction of their comparisons and the associated figures (e.g., ppe27 mutant vs wt).
- The authors have appropriately addressed my comment about the GSEA section.
- The authors have appropriately addressed the cytokine significance issue.
- The authors have appropriately clarified the four DEGs and the issue with gene duplication regarding JTY_3135 in their response and in their manuscript (e.g., line 311-314).
- The authors have improved the reproducibility of their incubation conditions and infection assay by providing the necessary details.
- The authors have included the requested CFU data and RT-PCR results along with NEW infection CFUs.
- The authors addressed the minor comments.
- The CFU data showing increased uptake and/or association of ppe27 vs wt bacteria at 4 hpi is very interesting. The authors' data with gentamicin also highlights the disadvantage of using antibiotics to "select" for intracellular bacteria.

Suggestions:

- Inclusion of the WGS data as a supplementary table would strengthen their conclusion because it is not included in the manuscript, other than Supplementary Figure 2 which shows the disrupted gene locus. For example, a table with the SNPs and other mutations from the wt and ppe27 mutant strains could be included (e.g., ppe27 compared to wt; both compared to the reference genome).

Response:

We appreciate the reviewer’s suggestion and have now included the WGS data in Supplementary Table 2. To improve genome coverage, we repeated the Nanopore long-read sequencing using the latest-generation R10.4.1 flow cell, which provides substantially improved sequencing accuracy compared with the previous version. In addition, we repeated the Miseq short-read sequencing at a higher sequencing depth. Based on the newly

generated sequencing data, whole-genome alignment, and subsequent variant analyses, we identified a 1-bp deletion in BCGΔppe27 within an intergenic region compared with wtBCG. We also confirmed the intended deletion region in BCGΔppe27. No additional differences were identified between the wtBCG and BCGΔppe27 strains (Supplementary Table 2B). In addition, we showed that the SNPs/InDels in wtBCG and BCG Δ ppe27 were identical, except for the aforementioned 1-bp intergenic deletion, when mapped to the reference genome (Supplementary Table 2C).

- The lack of a complement strain remains a pressing issue. The authors are correct that complementation can be problematic and difficult in Mycobacterium tuberculosis (MTB); however, there are numerous studies that have complemented MTB genes, regardless of the effects of the surrounding DNA sequence (i.e., genomic context). While the overexpression of ppe27 in Mycobacterium smegmatis led to increased production of TNFa, the same may not be observed in MTB. Furthermore, there are methods of controlling and/or titrating the expression of genes in MTB that may help to prevent overexpression, if it an issue (e.g., Tet- or nitrile-inducible promoter). Addressing this issue would greatly improve the study's validity.

Response:

We thank the reviewer for this insightful comment. In our study, the BCGΔppe27 mutant was generated by homologous recombination using a system based on the co-expression of mycobacteriophage proteins Che9c gp60 and gp61 from the pJV53 plasmid, as described in Materials and Methods. Although this system is highly efficient for replacing the target gene with a hygromycin-resistance cassette, the presence of the pJV53 plasmid in the bacteria complicates the selection of suitable complementation vectors.

Nevertheless, we fully agree that a complementation experiment using an inducible promoter, such as a Tet- or nitrile-inducible promoter, would be valuable for controlling PPE27 expression at a defined level and for addressing potential effects associated with gene overexpression. Although we were unable to perform this experiment in the current study, we appreciate the reviewer’s suggestion and will consider this approach in our future studies. Also, because there remains some uncertainty as to whether the observed phenomenon is solely attributable to PPE27, we have toned down our conclusions and emphasized the lack of complementation as a weakness of our study (line 431-437).

Reviewer #2 (Comments for the Author):

I appreciate the authors' efforts to revise the manuscript. The revised version is improved by the addition of culture and infection-preparation details, clarification of DEG comparison directions, additional RT-qPCR validation including IFN-related genes, JTY_3006 validation, CFU data, and more transparent statistical descriptions. The central observation that BCG Δ ppe27 infection is associated with enhanced inflammatory and IFN-related responses in THP-1-derived macrophages is generally supported by the presented RNA-seq, RT-qPCR, and cytokine data.
However, several important issues remain and should be addressed before publication.

1. The lack of a complemented strain remains a major limitation. The authors' explanation that complementation may be difficult because PPE27 expression requires native regulation is understandable, but not fully convincing. In the PE/PPE field, multiple previous studies, including work on PPE51/PE19, PPE51-mediated nutrient uptake, PE15/PPE20, and other PE/PPE proteins, have generated complemented strains or used functional complementation approaches to support causality (PMID: 32139546,32138343, 36441815, 42031738). Therefore, the general argument that PE/PPE complementation is unreliable or infeasible is too broad. If the authors cannot provide a complemented strain, they should explicitly acknowledge that causal attribution to ppe27 remains incomplete.

Response:
We sincerely thank the reviewer for this important comment and appreciate the suggested examples of complementation studies.
We agree that a complementation study is necessary for establishing the causal role of PPE27 in the observed phenotypes. Since we are unable to perform this experiment in the current study, we have revised our conclusions and explicitly acknowledged that the causal relationship between PPE27 and the observed phenotypes cannot be established without a complementation study. Specifically, we have added this statement to the final paragraph of the Discussion (lines 431-437) and toned down our conclusions, as described in our response to Reviewer 2's Comment 3.

2. WGS is useful but cannot substitute for complementation. The revised manuscript states that long- and short-read WGS confirmed the intended 476-bp deletion and did not identify off-target mutations. However, the manuscript does not provide sufficient detail on

sequencing depth, minor-variant thresholds, structural-variant calling, copy-number analysis, or how repetitive PE/PPE regions were resolved. This is particularly important because PE/PPE loci are repetitive and difficult to analyze, and low-frequency variants or structural changes may not be confidently excluded. The authors should soften statements implying that WGS fully rules out other genetic explanations.

Response:

We thank the reviewer for this constructive comment. To improve genome coverage, we repeated the Nanopore long-read sequencing using the latest-generation R10.4.1 flow cell, which provides substantially improved sequencing accuracy compared with the previous version. In addition, we repeated the Miseq short-read sequencing at a higher sequencing depth. We have now added the details of sequencing statistics, including sequencing depth and variant detection threshold (Supplementary Table 2A). We performed de novo assembly using both long- and short-read sequencing data and performed variant analyses to compare the differences between wtBCG and BCGΔppe27: Nanopore reads longer than 6,000 bp were used for de novo genome assembly with Flye v2.9.6. The resulting contigs were refined by incorporating sequence information from local assemblies of MiSeq short reads. Sequence differences between the wild-type and PPE27-deleted BCG strains were assessed by whole-genome alignment using MUMmer v3.23 (Materials and Methods).
Within this approach, we identified only a 1-bp deletion in BCGΔppe27 within an intergenic region compared with wtBCG. We also confirmed the intended deletion region in BCGΔppe27. No additional structural variants were identified by long-read sequencing, and no additional short variants were detected within our approach.

Although our WGS analyses did not identify any additional intragenic or structural variants, we acknowledge that other differences between the two strains cannot be entirely excluded. Therefore, we have limited our statements to those directly supported by the data.

"Long- and short-read sequencing confirmed the intended genetic modifications at the ppe27 locus, including the precise 476-bp deletion (Supplementary Figure 2A–2B). Using the combined long- and short-read approach (Supplementary Table 2A), only a 1-bp difference in a G homopolymer (37 nt versus 36 nt) was identified in the intergenic region between JTY_RS22225 and JTY_RS20995 (AP010918), in addition to the expected structural differences associated with the construction of the PPE27-deletion mutant; no other variants were identified between the two genomes (Supplementary Table 2B). Furthermore, reference-genome mapping identified the same set of SNPs/InDels in wtBCG

and BCG Δppe27, except for this 1-bp intergenic deletion (Supplementary Table 2C).” (lines 183-191)

3. The conclusions remain too strong. Statements such as "PPE27 is a negative regulator of host inflammatory responses," "PPE27 is not just an antigen but an active immune-modulating virulence factor," and "loss of a single PPE molecule causes a clear phenotype" should be revised. More appropriate wording would be: "ppe27 deletion is associated with enhanced macrophage inflammatory responses" or "PPE27 may contribute to modulation of host inflammatory responses."

Response:

We sincerely thank the reviewer for this valuable suggestion. We agree that the original wording may have overstated the conclusions that can be drawn from our data. Accordingly, we have carefully revised the relevant statements throughout the manuscript to more appropriately reflect the limitations of the current study (lines 54-56; 70-72).

4. The term "macrophage-associated bacterial RNA" should be used consistently. The authors appropriately changed much of the terminology from "intracellular" to "macrophage-associated," but some wording still implies a strictly intracellular bacterial population, such as "within host cells," "remove non-internalized bacilli," "bacteria residing in the host cells," and "internalized more efficiently." Because tightly surface-adhered bacteria cannot be excluded, these phrases should be revised.

Response:

We appreciate the reviewer’s suggestion. We have accordingly revised the wordings in line 277-278, line 334-335, line 538, and line 542.

5. The new CFU data are useful, especially the finding that bacterial burdens are comparable at 24 hpi. However, the higher Δppe27 burden at 4 hpi indicates that the two strains are not taken up or retained identically at early time points. Even if burdens are similar at 24 hpi, the host transcriptome at 24 hpi may still reflect different early exposure histories, different bacteria-per-cell distributions, or altered attachment/internalization dynamics. The authors should explicitly state this limitation. The conclusion should be framed as macrophage responses associated with Δppe27 infection under the tested conditions, rather than as a direct effect of PPE27 loss independent of bacterial uptake dynamics.

Response:

We thank the reviewer for these thoughtful comments. Accordingly, we have revised our descriptions in lines 385-393.

6. Only four DEGs were detected between wtBCG and BCGΔppe27 under macrophage-associated conditions, and two of the initially prominent candidates appear to be affected by multi-mapping. The validation of JTY_3006 is useful, but the link between PPE27, JTY_3006, SAM-dependent methyltransferase activity, and host epigenetic regulation remains speculative. The manuscript should clearly state that JTY_3006 is a candidate transcriptional change and that no direct evidence currently connects JTY_3006 to host epigenetic modification or the observed inflammatory phenotype.

Response:

We thank the reviewer for this suggestion and have accordingly added the statement in lines 427-430.

7. The revised Methods state that batch effects were accounted for by including a batch parameter in CLC Genomics Workbench. Please provide more detail on the statistical model/design formula, normalization method, whether the infection-day batch was paired across groups, and how the small sample size was handled. For GSEA, please specify the exact ranking metric, FDR threshold used to define significance, and whether all plotted pathways are significant or selected for visualization.

Response:

We thank the reviewer for carefully checking the methodological details.

Regarding CLC Genomics Workbench

・Statistical model/design formula

CLC Genomics Workbench includes a “Differential Expression for RNA-Seq” tool that uses a generalized linear model (GLM), assuming that read counts follow a negative binomial distribution. TMM normalization, a widely used method for DEG analysis, was applied to account for differences in library composition across samples. Each infection-day batch included samples from all experimental groups, and infection day was included as a covariate in the GLM to account for batch effects. These details have been added to Materials and Methods.

・Statistical handling when the sample size is small

Genomics Workbench (GWB) does not use a separate statistical model specifically for small sample sizes. However, when the number of replicates is small, the estimation of gene-wise dispersion relies more heavily on information from other genes with similar mean expression

levels.

We agree that the small sample size (n = 3 per group) limits statistical power and therefore warrants cautious interpretation of the differential expression results. However, when the number of replicates is small, gene-wise dispersion estimates in CLC Genomic Workbench are stabilized by borrowing information from genes with similar mean expression levels, based on the assumption that such genes tend to have similar dispersions. This approach helps improve the stability of dispersion estimates when only a limited number of biological replicates are available.

Regarding GSEA

The exact ranking metric was the *t*-test statistic (line 591). An FDR q-value < 0.05 was used as the threshold for statistical significance, and this information has now been added to lines 592-593 and the figure legend (line 905). All plotted pathways shown in the enrichment plots in Figure 3 were significant based on this criterion.

Re: Spectrum00915-26R2 (Deletion of *ppe27* in *Mycobacterium bovis* BCG augments macrophage immune responses during infection)

Dear Dr. Naoto Keicho:

Your manuscript has been accepted, and I am forwarding it to the ASM production staff for publication. Your paper will first be checked to make sure all elements meet the technical requirements. ASM staff will contact you if anything needs to be revised before copyediting and production can begin. Otherwise, you will be notified when your proofs are ready to be viewed.

Data Availability: ASM policy requires that data be available to the public upon online posting of the article, so please verify all links to sequence records, if present, and make sure that each number retrieves the full record of the data. If a new accession number is not linked or a link is broken, provide production staff with the correct URL for the record. If the accession numbers for new data are not publicly accessible before the expected online posting of the article, publication may be delayed; please contact ASM production staff immediately with the expected release date.

Publication Fees: For information on publication fees and which article types have charges, please visit our website. We have partnered with Copyright Clearance Center (CCC) to collect author charges. If fees apply to your paper, you will receive a message from no-reply@copyright.com with further instructions. For questions related to paying charges through RightsLink, please contact CCC at ASM_Support@copyright.com or toll free at +1-877-622-5543. CCC makes every attempt to respond to all emails within 24 hours.

ASM Membership: Corresponding authors may join or renew ASM membership to obtain discounts on publication fees. Need to upgrade your membership level? Please contact Customer Service at Service@asmusa.org.

Embargo Policy: A press release may be issued as soon as the manuscript is posted on the Spectrum Latest Articles webpage. The corresponding author will receive an email with the subject line "ASM Journals Author Services Notification" when the article is available online.

Cover Image Submissions: If you would like to submit a potential Cover Image, please email a file and a short legend to spectrum@asmusa.org. Please note that we can only consider images that (i) the authors created or own and (ii) have not been previously published. By submitting, you agree that the image can be used under the same terms as the published article. Image File requirements: TIF/EPS, 7.5 inches wide by 8.25 inches tall (at least 2,250 pixels wide by 2,475 pixels tall), minimum 300 dpi resolution (600 dpi preferred), RGB, and no figure elements, e.g., arrows or panel labels. The legend should be a short description of the image, 1-2 sentences recommended. Please use the ASM Journals Cover Image Sizing Tool (https://journals.asm.org/pb-assets/html/asm-cover-sizing-tool.html) to ensure that your proposed cover image meets our size requirements.

The ASM Journals program strives for constant improvement in our submission and publication process. Please tell us how we can improve your experience by taking this quick Author Survey.

Thank you for submitting your paper to Spectrum.

Sincerely,
Sladjana Prisic
Editor
Microbiology Spectrum
