# Supplementary material for: Deletion of ppe27 in Mycobacterium bovis BCG augments macrophage immune responses during infection
Source: Microbiol Spectr. 2026 Oct 6;14(11):e00915-26. doi: 10.1128/spectrum.00915-26 (PMC13729191; doi:10.1128/spectrum.00915-26)

Supplementary Figure 1. Illustration of the disrupted *ppe27* sequence. Ins-F1/Ins-R1 primers were employed to delete 476 bp (marked in blue), and the hygromycin gene was inserted via homologous recombination as described in the Materials and Methods. Red asterisks denote hydrogen-bonding interactions between PE18 and PPE27, whereas the green asterisk denotes hydrogen-bonding interactions between EspG5 and PPE27, as reported recently (Ehtram et al., 2025). This deletion resulted in the disruption of the hydrogen bonding of PPE27 to EspG5 and PE18. Therefore, the mutated PPE27 protein is non-functional, and cannot be transported or secreted out of the membrane.

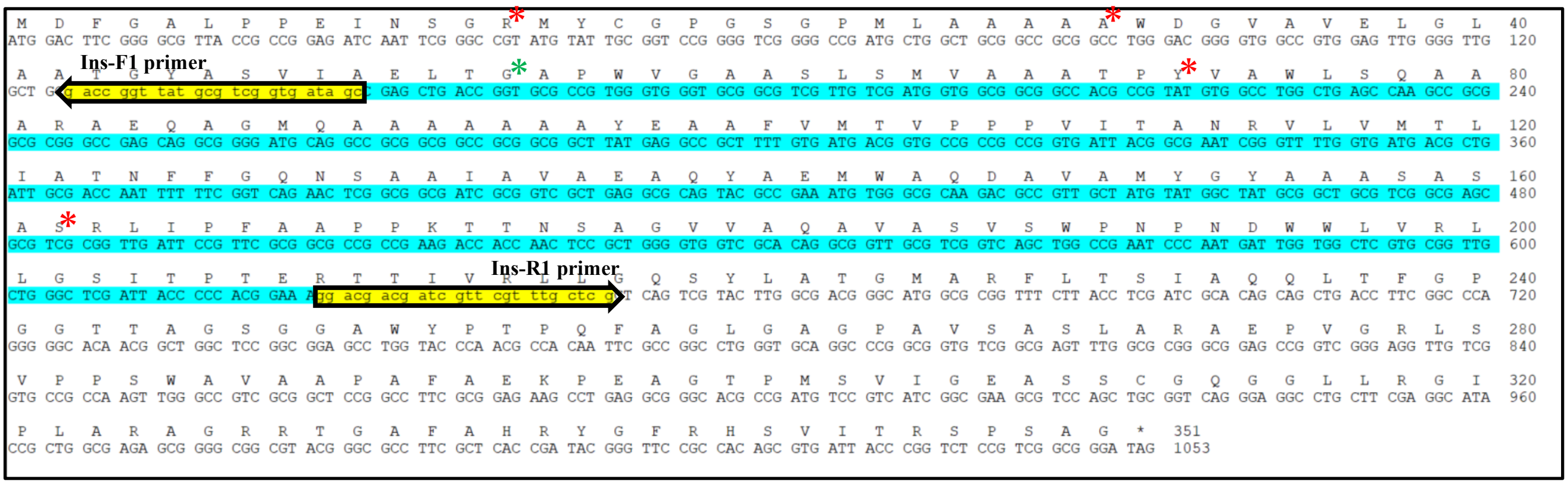

Supplementary Figure 2. Whole-genome sequencing of bacterial DNA. (A) Long-read sequencing by Oxford Nanopore Technologies. The full length of *ppe27* is 1,053 bp and mutagenesis disrupted *ppe27* by deleting 476 bp within the gene. (B) Short-read sequencing using Illumina MiSeq. The deleted region is marked by a red rectangle. The yellow lines indicate reads with multiple hits (non-specific matches).

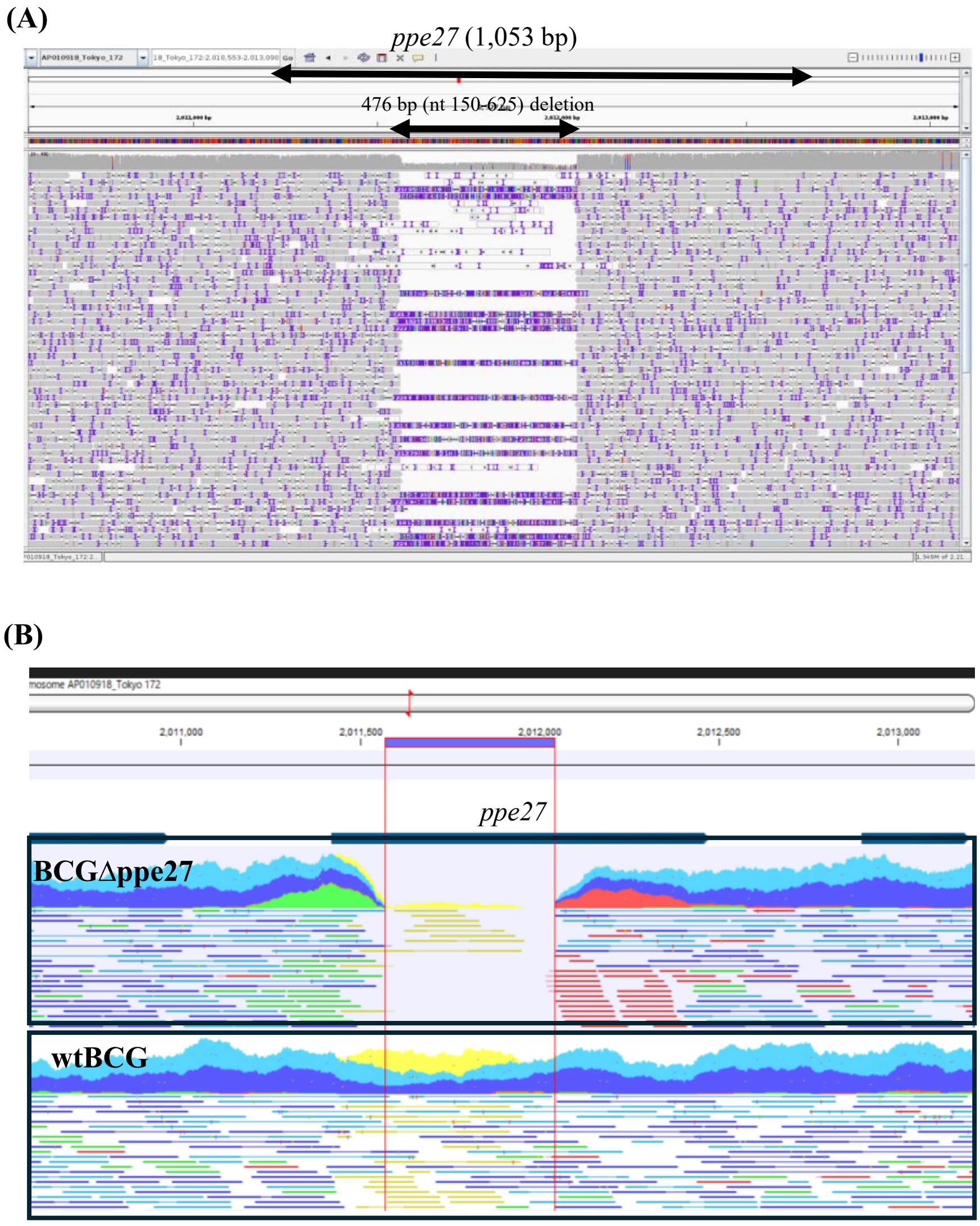

Supplementary Figure 3. *JTY_3006* encodes a 286-amino acid protein. Alignment of the amino acid sequence of JTY_3006 revealed 100% identity to the protein encoded by the orthologous gene Rv2990c in *Mycobacterium tuberculosis* H37Rv (AL123456). The functional product of this protein is recognized as class I SAM-dependent methyltransferase. Alignment was generated using Clustal W.

```
CLUSTAL 2.1 multiple sequence alignment

Mtb_H37Rv_Rv2990c          MCVTWAEMPKIAALIRHIEDLHARHGRSYILRAGISSLFRYIEGVHGERPWGTVLDAGTG
BCG_Tokyo172_JTY3006       MCVTWAEMPKIAALIRHIEDLHARHGRSYILRAGISSLFRYIEGVHGERPWGTVLDAGTG
                           ************************************************************

Mtb_H37Rv_Rv2990c          VKSLQWIQTLPTERWTAVTAARSLADKTRAALGSAMRPQDRLLVGNWVDDSLLAGETFDT
BCG_Tokyo172_JTY3006       VKSLQWIQTLPTERWTAVTAARSLADKTRAALGSAMRPQDRLLVGNWVDDSLLAGETFDT
                           ************************************************************

Mtb_H37Rv_Rv2990c          ILVDYLVGAIEGFAPYWQDRVFERLRPHLADHGRLYLVGLEPYVQFEPETESGKIIWEIG
BCG_Tokyo172_JTY3006       ILVDYLVGAIEGFAPYWQDRVFERLRPHLADHGRLYLVGLEPYVQFEPETESGKIIWEIG
                           ************************************************************

Mtb_H37Rv_Rv2990c          RVRDACLLLAGERPYREFPLDWMLGRLGLAGFRILEARRFPIRYRARYVNGQLNMCLARI
BCG_Tokyo172_JTY3006       RVRDACLLLAGERPYREFPLDWMLGRLGLAGFRILEARRFPIRYRARYVNGQLNMCLARI
                           ************************************************************

Mtb_H37Rv_Rv2990c          ERFSSNGLGMAMRAYVEELRARALQLNERQDGLWHGNDYVIAVEPM
BCG_Tokyo172_JTY3006       ERFSSNGLGMAMRAYVEELRARALQLNERQDGLWHGNDYVIAVEPM
                           **********************************************
```

Supplementary Figure 4. Validation of macrophage-associated bacterial DEG, *JTY_0110,* by RT-qPCR.

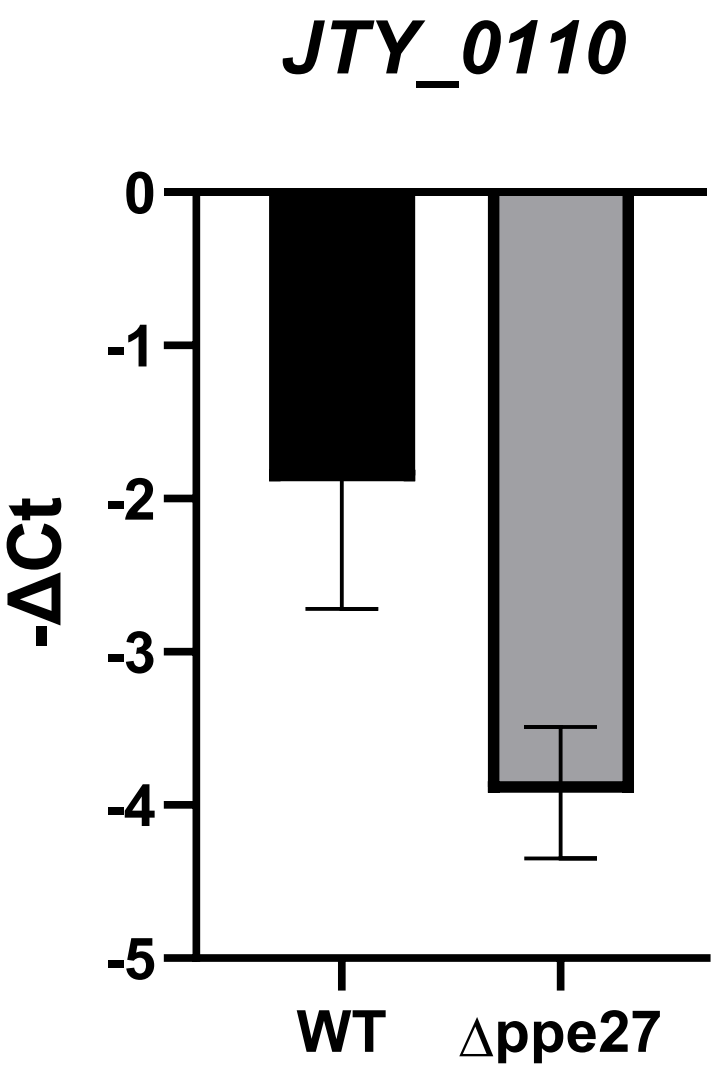

Supplementary Figure 5. Macrophage-associated bacterial burden at 24 h post-infection determined by CFU assay.

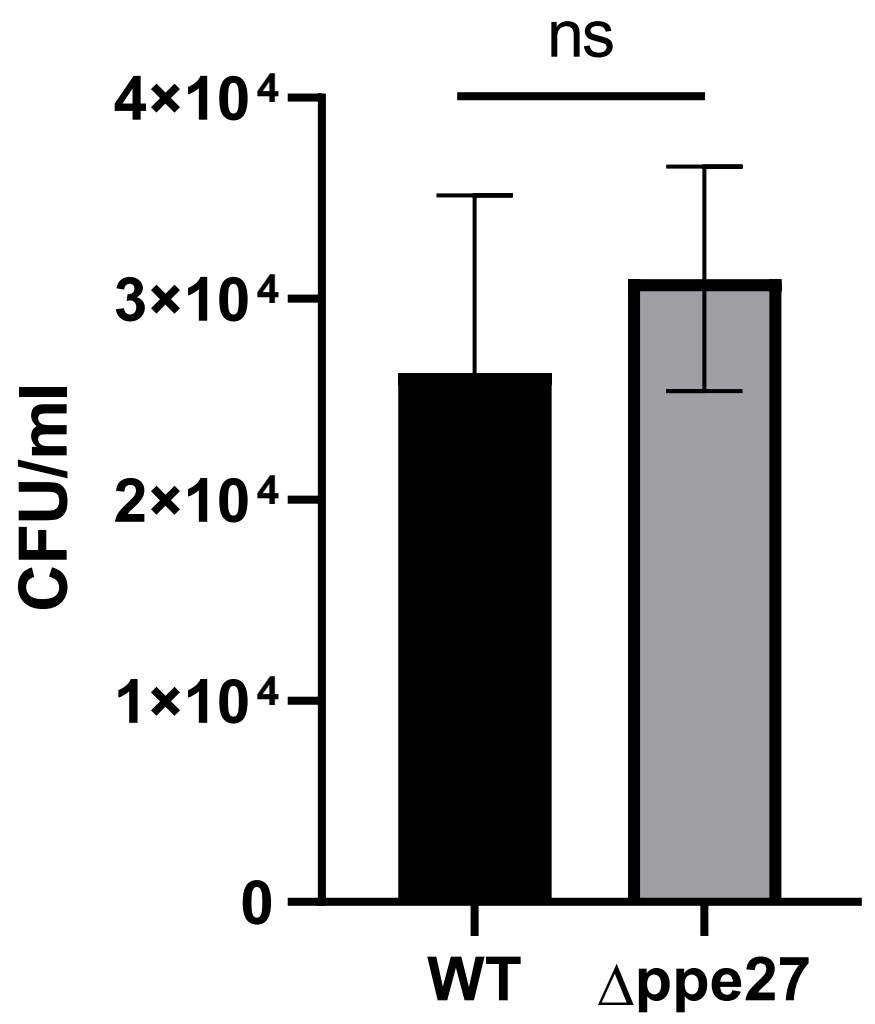

Supplementary Figure 6. Bacterial uptake efficiency. Macrophage-associated bacterial burden was quantified at 4h post-infection by CFU assay.

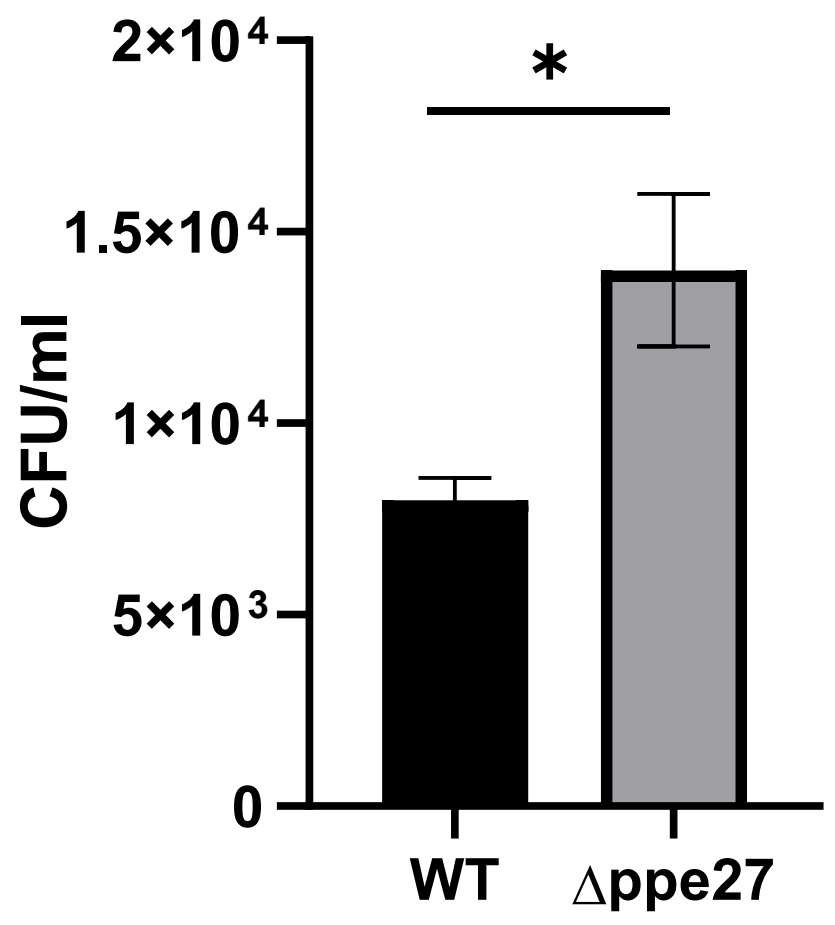

Supplement: Supplementary file 2 [file spectrum.00915-26-s0001.pdf]
